# Targeting lysine-specific demethylase 1 (KDM1A/LSD1) impairs colorectal cancer tumorigenesis by affecting cancer cells stemness, motility, and differentiation

**DOI:** 10.1038/s41420-023-01502-1

**Published:** 2023-06-29

**Authors:** Annamaria Antona, Giovanni Leo, Francesco Favero, Marco Varalda, Jacopo Venetucci, Stefania Faletti, Matilde Todaro, Eleonora Mazzucco, Enrica Soligo, Chiara Saglietti, Giorgio Stassi, Marcello Manfredi, Giuliana Pelicci, Davide Corà, Guido Valente, Daniela Capello

**Affiliations:** 1 Department of Translational Medicine, Centre of Excellence in Aging Sciences, Università del Piemonte Orientale, Via Solaroli 17, 28100 Novara, Italy; 2 Center for Translational Research on Autoimmune and Allergic Diseases, Department of Translational Medicine, Università del Piemonte Orientale, Corso Trieste 15/A, 28100 Novara, Italy; 3 Department of Experimental Oncology, IRCCS, European Institute of Oncology, Via Adamello 16, 20139 Milano, Italy; 4 Department of Health Promotion, Mother and Child Care, Internal Medicine and Medical Specialties (PROMISE), University of Palermo, Piazza delle Cliniche 2, 90127 Palermo, Italy; 5 Pathology Unit, Ospedale Sant’Andrea, Corso Mario Abbiate 21, 13100 Vercelli, Italy; 6 Department of Surgical, Oncological and Stomatological Sciences, Università di Palermo, Via del Vespro 131, 90127 Palermo, Italy

**Keywords:** Cancer stem cells, Colorectal cancer, Protein-protein interaction networks, RNA sequencing

## Abstract

Among all cancers, colorectal cancer (CRC) is the 3rd most common and the 2nd leading cause of death worldwide. New therapeutic strategies are required to target cancer stem cells (CSCs), a subset of tumor cells highly resistant to present-day therapy and responsible for tumor relapse. CSCs display dynamic genetic and epigenetic alterations that allow quick adaptations to perturbations. Lysine-specific histone demethylase 1A (KDM1A also known as LSD1), a FAD-dependent H3K4me1/2 and H3K9me1/2 demethylase, was found to be upregulated in several tumors and associated with a poor prognosis due to its ability to maintain CSCs staminal features. Here, we explored the potential role of KDM1A targeting in CRC by characterizing the effect of KDM1A silencing in differentiated and CRC stem cells (CRC-SCs). In CRC samples, KDM1A overexpression was associated with a worse prognosis, confirming its role as an independent negative prognostic factor of CRC. Consistently, biological assays such as methylcellulose colony formation, invasion, and migration assays demonstrated a significantly decreased self-renewal potential, as well as migration and invasion potential upon KDM1A silencing. Our untargeted multi-omics approach (transcriptomic and proteomic) revealed the association of KDM1A silencing with CRC-SCs cytoskeletal and metabolism remodeling towards a differentiated phenotype, supporting the role of KDM1A in CRC cells stemness maintenance. Also, KDM1A silencing resulted in up-regulation of miR-506-3p, previously reported to play a tumor-suppressive role in CRC. Lastly, loss of KDM1A markedly reduced 53BP1 DNA repair foci, implying the involvement of KDM1A in the DNA damage response. Overall, our results indicate that KDM1A impacts CRC progression in several non-overlapping ways, and therefore it represents a promising epigenetic target to prevent tumor relapse.

## Introduction

Colorectal cancer (CRC) represents the third most common cancer worldwide and the second cause of oncological death [1]. The global burden of CRC is expected to increase due to the growth and aging of the population, whereas screening programs and early diagnosis decreased significantly CRC mortality [2]. However, only about 20% of CRC patients are diagnosed at an early stage, in which the 5-year relative survival rate is 91%, whereas the 5-year survival drops to 12% in CRC patients diagnosed with stage IV disease [3]. Thus, CRC treatment remains an existing oncological challenge.

Cancer stem cells (CSCs) are crucial players in tumor initiation and development, leading to intratumoral heterogeneity, conventional therapy resistance, and recurrence [4]. Efforts have been made to target CSCs or to prevent signals from the microenvironment that regulate stem cell features, including self-renewal, differentiation, and apoptosis evasion [5]. Yet, since CSCs and normal SCs share similarities, the specificity of CSCs-targeting treatments remains a current issue for their introduction into clinical practice [6].

Compelling evidence supports epigenetic targeting in CSCs as an attractive therapeutic option due to the relevant role of epigenetic deregulation in promoting tumorigenesis and cellular plasticity [7]. To date, several epigenome-targeting drugs have been approved or are currently under clinical trials, including inhibitors of DNA methyltransferases (DNMTi) and histone deacetylase (HDACi) [8]. However, different epigenetic inhibitors generated unwanted effects, and acquired resistance to epi-drugs is becoming a therapeutic challenge [9]. Therefore, the combination of DNMTi and HDACi, and the association of epi-drugs with cytotoxic agents, have been extensively investigated [9, 10].

KDM1A (also known as LSD1) is a member of the flavin-dependent amine oxidase protein family and was the first histone demethylase discovered [11]. KDM1A associates with different partners and acts as a co-repressor when interacting with COREST or NURD to demethylate H3K4me1/2 [12], or as a co-activator when interacting with androgen or estrogen receptor to demethylate H3K9me1/2 [13]; additionally, KDM1A has several non-histone targets [14]. Numerous studies underlined the substantial contribution of KDM1A during carcinogenesis. Indeed, KDM1A is highly expressed both in hematological malignancies, including acute myeloid leukemia [15], and solid tumors, such as CRC, prostate, lung, brain, and breast cancers [16]. KDM1A overexpression is usually associated with poor prognosis, and it has been proposed as a biomarker in different tumors, including CRC [17, 18]. Accordingly, pharmacologic KDM1A inhibition demonstrated efficacy in reducing tumor growth and progression in vitro and in vivo [19, 20]. Importantly, KDM1A plays a pivotal role in both normal SCs and CSCs [21]. Particularly, KDM1A is involved in stemness maintenance of glioblastoma [22], hepatocellular carcinoma [23], breast cancer [24], and leukemic [15] SCs.

Notably, a contribution of KDM1A in the maintenance of CRC stem-like cells (CRC-SCs) features has been recently demonstrated [25]. However, since KDM1A’s involvement in CRC-SCs maintenance and plasticity remains poorly characterized, further studies are warranted. Herein, starting from a retrospective cohort analysis, and performing a deep investigation of the phenotypic and molecular effects of KDM1A silencing in human primary tumor-derived CRC-SCs and, for comparison, in adherent cells, we investigated KDM1A as a possible prognostic and therapeutic target in CRC.

## Results

### KDM1A expression predicts a worse prognosis in CRC patients

**Fig. 1:**
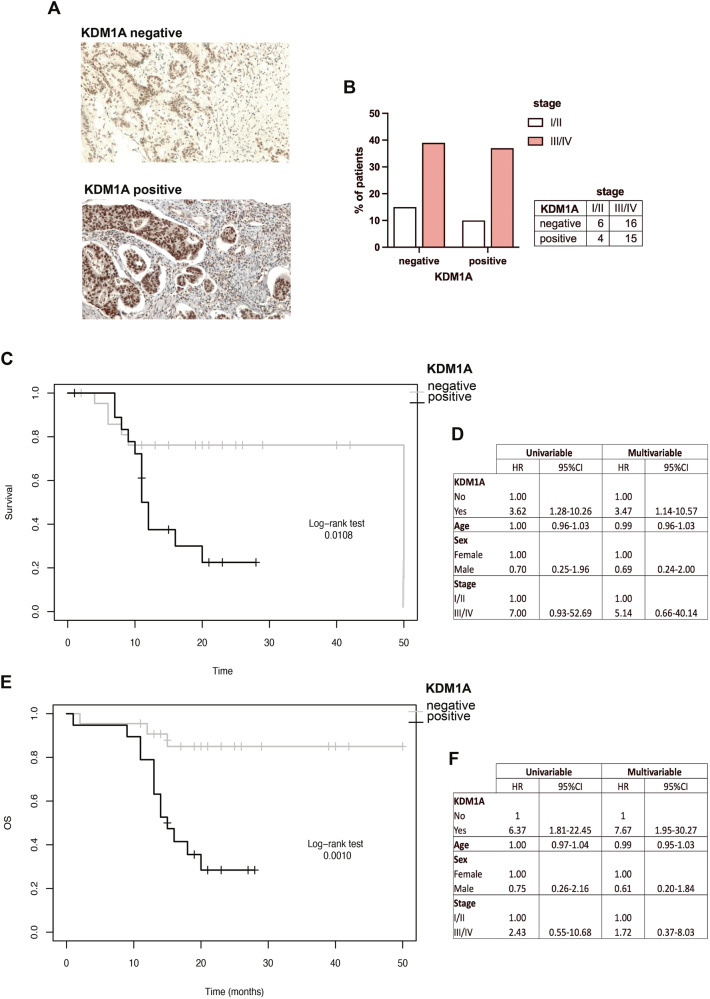
KDM1A expression foresees a worse prognosis in CRC. Immunohistochemistry staining of KDM1A in normal and CRC tissues, based on nuclear staining intensity score multiplied by the percentage of KDM1A-positive cells (<80 negative; >80 positive) (**A**). Chi-square analysis displaying the correlation between KDM1A and tumor stage in CRC patients (**B**). Kaplan–Meier curves with log-rank survival analyses showing that KDM1A expression is associated with reduced progression-free survival (PFS) and overall survival (OS) (**C**, **E**). Univariable and multivariable Cox proportional hazards regression analysis showing hazard ratio (HR) and 95% confidence interval (CI) for each covariate (**D**, **F**), *p*-values < 0.05 were considered statistically significant.

KDM1A has been reported to be overexpressed in CRC cells but studies assessing the relationship between KDM1A up-regulation and prognosis are controversial [26, 27]. Here, we evaluated KDM1A expression in CRC patients (Fig. 1A) and its association with overall survival (OS) and progression-free survival (PFS). A total of 41 CRCs derived from patients were retrospectively analyzed (31 males and 10 females) with an average age of 66.4 +/− 13.4 years. Five out of forty-one (7.3%) cancers were stage I, 5/41 (12.2%) stage II, 28/41 (68.3%) stage III, and 3/41 (40.7%) stage IV based on the AJCC staging system [28] (Supplementary Table 1).

KDM1A was expressed in 19/41 cancers (46.3%) and its expression did not significantly associate with tumor stage (chi-square test; *p* = 0.644) (Fig. 1B). Kaplan–Meier Log Rank survival analyses were performed to analyze the expression of the KDM1A in clinical prognosis. KDM1A expression was significantly associated with a shorter PFS (*p* = 0.0108) (Fig. 1C) and OS (*p* = 0.001) (Fig. 1E).

We used the Cox proportional hazards regression method to investigate the effect of covariates such as age, sex, tumor stage, and KDM1A expression on PFS and OS; we obtained a hazard ratio (HR) and 95% confidence interval (CI) for each covariate (univariable and multivariable) (Fig. 1D, F). Results showed that KDM1A expression implies a poorer prognosis on PFS (HR: 3.47, 95% CI: 1.14–10.57, *p* = 0.0289) and a worse OS (HR: 7.67, 95% CI: 1.95–1.03, *p* = 0.0036), while the remaining covariates (age, sex, tumor stage) showed no statistical significance on PFS and OS (all *p* > 0.05). Therefore, these findings support the role of KDM1A as an independent negative prognostic factor of CRC.

### Knockdown of KDM1A impairs CRC cells viability, induces cells cycle arrest, and results in a defective DNA damage response

**Fig. 2:**
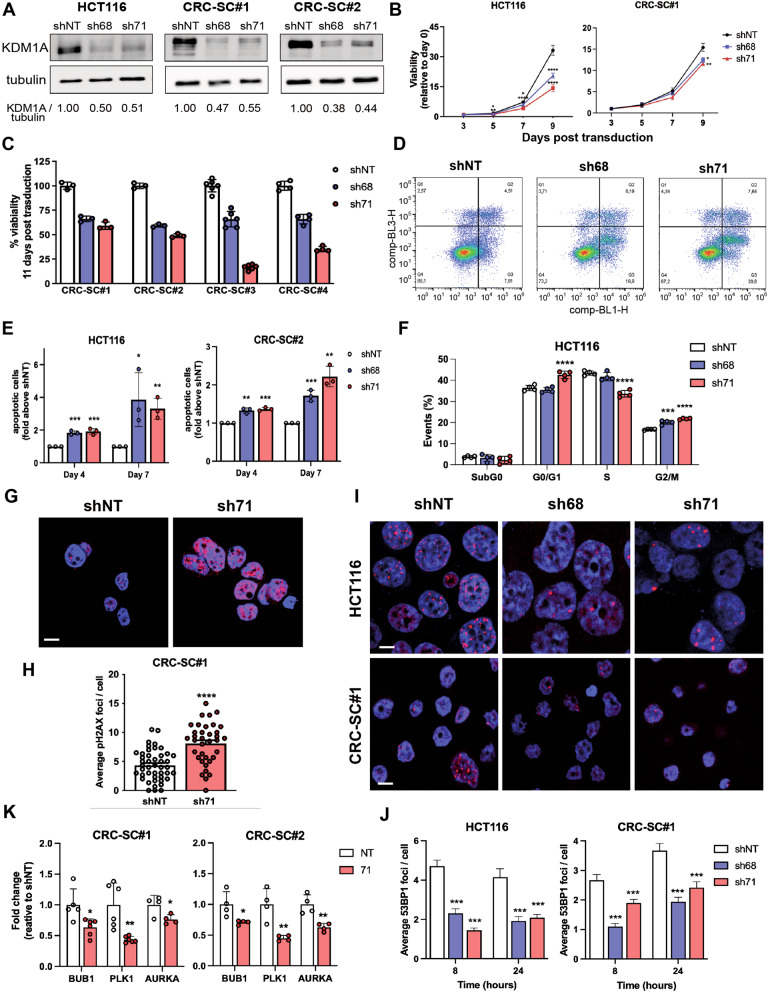
KDM1A knockdown induces apoptosis, cell cycle arrest, and defective DDR. Western blots showing KDM1A protein silencing upon shRNA-mediated transduction of HCT116 cells and CRC-SCs with shNT and the shKDM1A (sh68 and sh71) on day 3 after transduction (protein quantification normalized on tubulin, relative to shNT) (**A**). Cell viability assay using CellTiter-Glo to measure ATP content in CRC cells on days 5, 7, and 9 after transduction. Data represent the mean ± SD of at least three independent experiments (**B**). Viability of CRC-SCs (*n* = 4) 11 days after transduction with shNT, sh68, and sh71 shRNA expressing lentiviruses. Data represent the mean ± SD of at least three independent experiments (**C**). Representative dots plots showing HCT116 cells distribution after AxV/PI staining (**D**). Graphs showing the analysis of apoptotic cells (AxV pos/PI neg) after KDM1A silencing in HCT116 and CRC-SC#1 cells on day 4 and day 7 after transduction. Data represent the mean ± SD of at least three independent experiments (**E**). Cell cycle distribution quantification (**F**) of transduced HCT116 cells by flow cytometry. The percentage of cells in sub G0, G0/G1, S, and G2 phases of the cell cycle was assessed 48 h after serum addition to starved transduced cells (day 2 after transduction). Data represent the mean ± SD of three independent experiments. For DDR investigation, cells (day 2 after transduction) were pretreated with oxaliplatin 10 μmol/L for 8 h to induce DSBs formation. After oxaliplatin removal cells were stained for nuclei (blue), 53BP1 (red), or pH2AX (red), and mounted on microscope slides 8- and 24 h following treatment removal. Pictures were acquired by confocal microscopy (63X magnification, scale bar 25 μm). Representative pictures (**G**, **I**). Graphs showing 53BP1 or pH2AX foci average number per cell (**H**, **J**). Data show the mean ± SEM of three independent experiments. RT-qPCR analysis of BUB1, PLK1, and AURKA in CRC-SC#1 and CRC-SC#2 KDM1A-silenced cells (**K**). Data represent the mean ± SD of at least three independent experiments. Student’s t-test was conducted (sh68 or sh71 versus shNT): *p < 0.05; **p < 0.01; ***p < 0.001; ****p < 0.0001.

KDM1A is overexpressed in several cancers, where it inhibits cell differentiation while enhancing cell proliferation and aggressiveness [14, 29]. Also, evidence reported the critical role of KDM1A in preserving tumor SCs compartment and, consequently, promoting tumor development [20, 21]. Thus, we investigated the role of KDM1A in CRC-SCs survival and proliferation at different time points. Stably transduced cells expressing two distinct KDM1A-targeting shRNA sequences (sh68 and sh71), or non-targeting shRNA (shNT) were obtained through lentiviral infection. Knockdown efficiency was validated by western blotting (Fig. 2A, Original Data File). Upon shRNA-induced knockdown of KDM1A, a moderate but significant decrease in cell viability was detected in all tested primary tumor-derived CRC-SCs (*n* = 4) and HCT116 cells, with more than 20% inhibition of cell viability over a timeframe of 9 days (Fig. 2B, C).

The decreased viability of KDM1A-silenced cells prompted us to investigate apoptosis and cell cycle. Apoptosis was assessed by Annexin V (AxV) and propidium iodide (PI) staining and flow cytometry analysis at 4 and 7 days after transduction. A significant increase of apoptotic cells (AxV pos/PI neg) was observed already on day 4, with more than a 2 to 3-fold increase on day 7 (Fig. 2D, E). Cell cycle analysis revealed arrest in the G2/M phase upon KDM1A silencing (Fig. 2F) in HCT116 cells. These results are consistent with prior studies reporting lower proliferation rates and cell-cycle arrest in the G2-M phase after KDM1A inhibition in lung adenocarcinoma and endothelial cells [30, 31].

Cell cycle checkpoints, coordinating cell cycle arrest and DNA repair, are frequently triggered by DNA damage [32]. KDM1A was reported to play a role in the DNA damage response (DDR) through its interaction with the E3 ubiquitin ligase RNF168 for 53BP1 recruitment to the sites of DNA damage [33, 34]. Thus, we explored KDM1A’s role in promoting DDR in CRC cells, by performing immunofluorescence analysis to evaluate the number of 53BP1 foci in KDM1A-silenced cells. Cells were treated for 8 h with oxaliplatin 10 µmol/L for the induction of DNA damage and double-strand break (DSB) formation. A significant increase of H2AX phosphorylation was observed 24 h after oxaliplatin treatment (Fig. 2G, H), whereas a sharp reduction of 53BP1 foci number was evident in both HCT116 and CRC-SC#1 KDM1A-silenced cells, compared with controls, at 8- and 24-h after oxaliplatin treatment (Fig. 2I, J).

When cells in the G2 phase are exposed to DNA damage, numerous critical mitotic regulators are repressed, including AURKA and PLK1 [35]. RT-qPCR analysis confirmed that, as reported by Dalvi et al., the role of KDM1A in cell-cycle progression is linked to the regulation of the crucial cell-cycle kinase PLK1 pathway, including BUB1, PLK1, and AURKA (Fig. 2K). These results suggest that, in the absence of KDM1A, the first steps of the DDR repair process are effective, whereas the recruitment of the entire DDR machine is defective, impairing DNA repair and cell cycle progression.

### KDM1A is required for CRC cells clonogenic potential maintenance and anoikis resistance

**Fig. 3:**
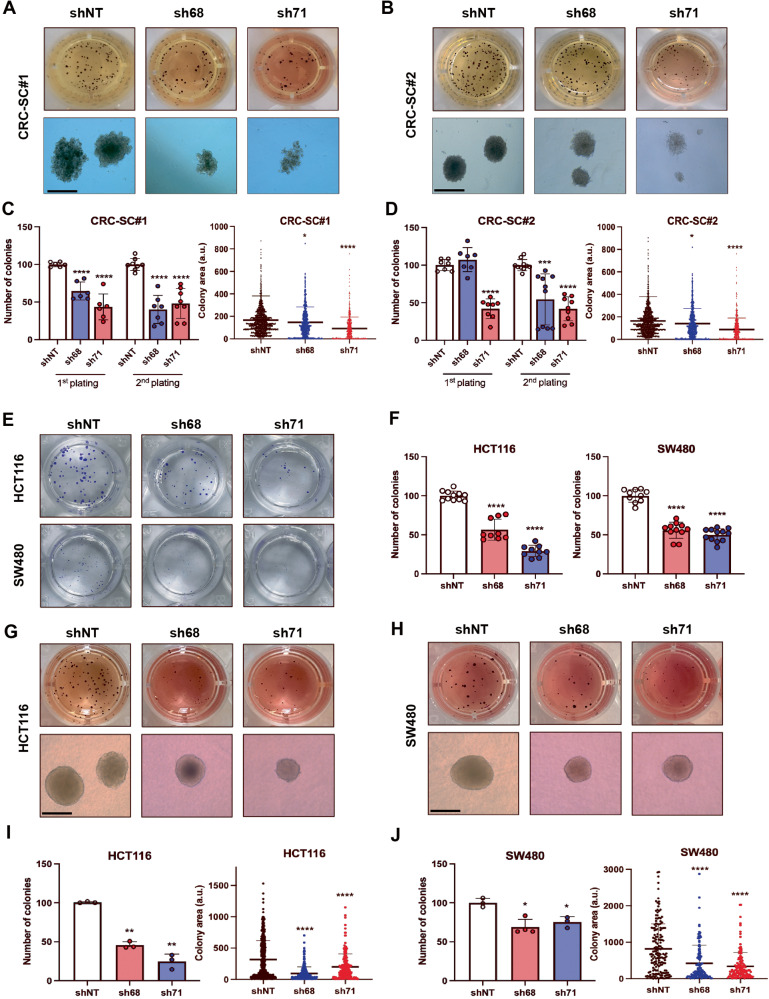
KDM1A silencing reduces CRC cells clonogenic potential and anoikis resistance. Cells were transduced with control shNT and shRNAs specific for KDM1A, sh68, and sh71. Representative pictures of the methylcellulose colony formation assay (CRC-SC#1 and CRC-SC#2) stained with MTT or acquired with a phase-contrast microscope (scale bar 250 μm) CRC-SC#1 (**A**) and CRC-SC#2 (**B**). Quantification of the number of colonies revealing a significant reduction in the clonogenic potential in CRC-SC#1 (**C**) and CRC-SC#2 (**D**). Data are presented as mean ± SD from three independent experiments. Representative pictures of the colony formation assay of HCT116 and SW480 cells stained with crystal violet (**E**). Quantification of the number of colonies revealing a significant reduction in the clonogenic potential (**F**). Data are presented as mean ± SD from three independent experiments. Representative pictures of transduced HCT116 (**G**), and SW480 (**H**) cells seeded in soft agar and stained with MTT. Quantification graphs showing a remarkable decrease in the number of colonies and their area in HCT116 (**I**), and SW480 (**J**) KDM1A-silenced cells. Data are presented as mean ± SD from at least three independent experiments. The number and area of the colonies were assessed by ImageJ software. Student’s t-test was conducted (sh68 or sh71 vs shNT): *p < 0.05; **p < 0.01; ***p < 0.001; ****p < 0.0001.

KDM1A has been reported to be required for the maintenance of both normal and neoplastic SCs [20, 21]. We, therefore, investigated the role of KDM1A in stemness and clonogenic potential of CRC cells. A specific 2-step methylcellulose clonogenic assay was performed using KDM1A-silenced or control CRC-SC#1 and CRC-SC#2 cells in order to evaluate stem cells enrichment and their clonogenic potential (Fig. 3A, B). Assessment of CRC-SCs clonogenicity revealed a significant reduction in the number of colonies in KDM1A-silenced samples, in both the first and second plating for CRC-SC#1 and the second plating for CRC-SC#2 (Fig. 3C, D).

Clonogenicity was also investigated by a 2D colony formation assay, that showed a nearly 50% reduction in the colony formation capacity of HCT116 and SW480 KDM1A-silenced cells (Fig. 3E, F). Afterward, the clonogenic potential and anoikis resistance were evaluated in a condition of adhesion-independent cell proliferation by soft agar assay. A reduction in the number of colonies by 50% and 30% in HCT116 and SW480 KDM1A-silenced cells, respectively, and a significant decrease in the colonies area, were observed (Fig. 3G–J). These results corroborate the role of KDM1A in stemness maintenance and resistance to anoikis, a type of cell death induced by the loss of contact with the extracellular matrix representing an obstacle for metastasis formation [36].

### KDM1A is required for CRC cells migration and invasion

**Fig. 4:**
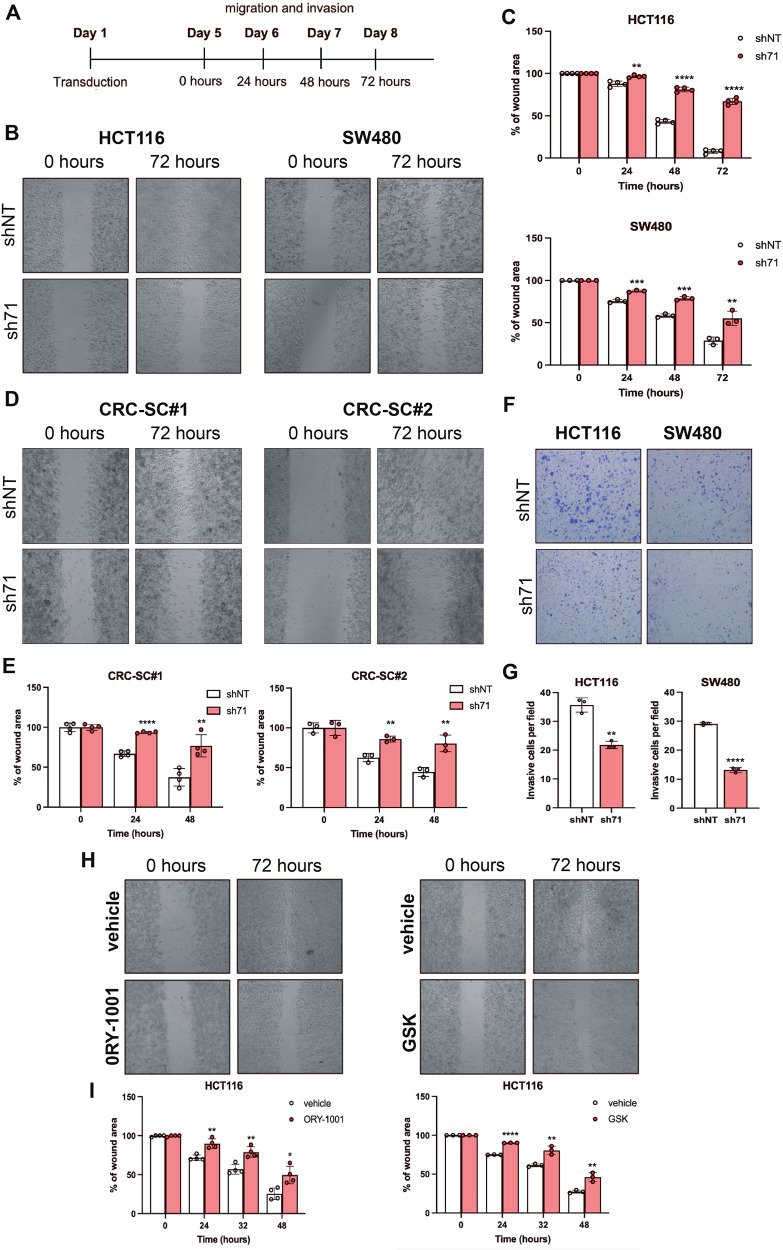
KDM1A targeting impairs CRC cells migration and invasion. Timeline of the investigation of the effect of KDM1A targeting on invasion and migration (**A**). Representative phase-contrast pictures of wound healing assays of HCT116 and SW480 cells (**B**). Data analysis of the migration potential of adherent KDM1A-silenced cells vs control cells (shNT). Data represent the mean ± SD of at three independent experiments (**C**). Representative phase-contrast pictures of wound healing assays of CRC-SC#1 and CRC-SC#2 (**D**). Data analysis of the migration potential of KDM1A-silenced CRC-SCs vs controls (shNT). Data represent the mean ± SD of at least three independent experiments (**E**). Representative phase-contrast pictures of transwell invasion assay in adherent CRC cells stained with DiffQuick (**F**). Data analysis of invading cells at 48 h. Data represent the mean ± SD of at least three independent experiments (**G**). Wound healing assay of HCT116 cells treated with the KDM1A inhibitors ORY-1001 (10μmol/L) and GSK2879552 (40 μmol/L); representative pictures (**H**) and data analysis (**I**) of the migration potential of adherent cells treated with KDM1A inhibitors vs vehicle. Data represent the mean ± SD of at least three independent experiments. The percentage of wound area was assessed by ImageJ software. Student’s t-test was conducted (sh68 or sh71 versus shNT): *p < 0.05; **p < 0.01; ***p < 0.001; ****p < 0.0001.

Immunohistochemistry conducted on the retrospective cohort of CRC samples demonstrated a positive association between KDM1A expression in primary tumors and metastatic disease. Moreover, our experiments showed that KDM1A silencing impairs anoikis resistance, suggesting that KDM1A strongly contributes to cell adhesion, migration, and invasion, required for metastases formation. We, therefore, evaluated HCT116, SW480, and CRC-SCs cells migration and invasion following KDM1A silencing at different time points (Fig. 4A). The wound-healing assay revealed a dramatic reduction in cell migration of both differentiated and stem-like cells after KDM1A silencing (Fig. 4B–E); besides, transwell invasion assay showed a significant reduction of the invasive potential of KDM1A-silenced cells (Fig. 4F, G). Notably, despite pharmacological targeting of KDM1A showed no significant acute cytotoxicity in several CRC cell lines (Supplementary Fig. 1), KDM1A inhibitors, such as ORY-1001 and GSK2879552, reduced cell migration at a clinically relevant dose (Fig. 4H, I). Our results underpin the pivotal role of KDM1A in promoting CRC metastatic potential.

### Transcriptomic analysis revealed the induction of a gene expression profile consistent with cell differentiation in CRC-SCs upon KDM1A-silencing

**Fig. 5:**
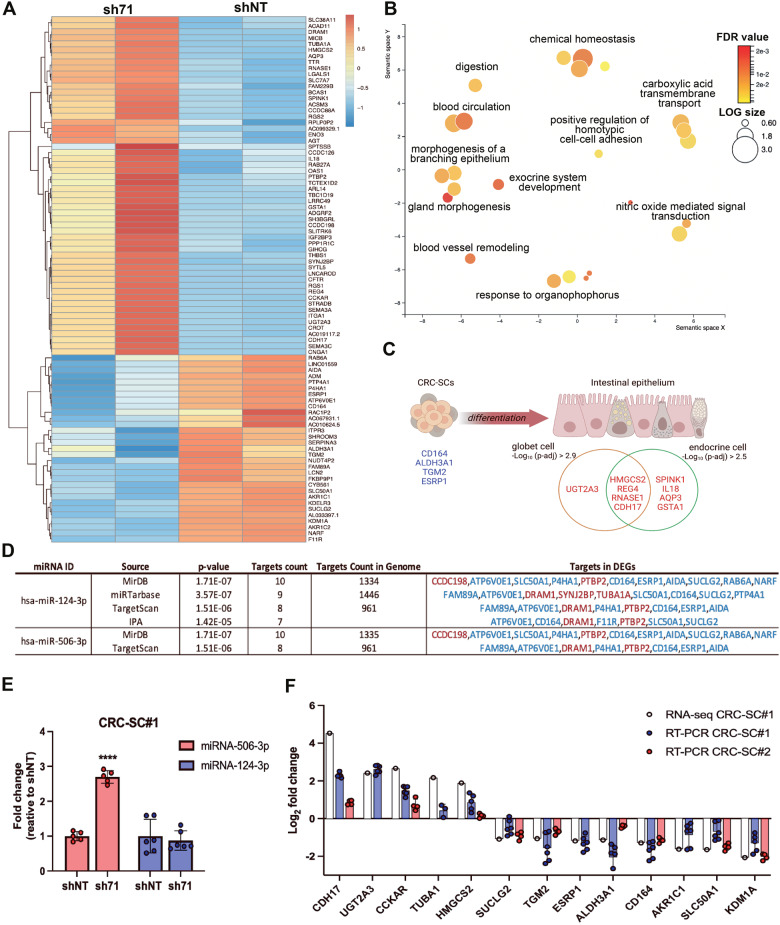
KDM1A-silenced CRC-SCs express differentiation markers. Heatmap showing the unsupervised hierarchical clustering of the 89 DEGs identified comparing CRC-SC#1 silenced cells (sh71) vs CRC-SC#1 control cells (shNT) (**A**). Semantic plot of the enriched biological processes (**B**). Schematic representation of deregulated genes associated with differentiation of intestinal cells (downregulated genes are displayed in blue, while upregulated genes are in red). Endocrine and goblet cells markers clustering was obtained through g:Profiler (Human Protein Atlas’s results). Created with BioRender.com (**C**). Prediction of upstream miRNA regulators and target DEGs by using available online tools and based on DEGs shown in (**A**) (downregulated genes are displayed in blue, while upregulated genes are in red) (**D**). RT-PCR expression of miRNA-124-3p and miRNA-506-3p in CRC-SC#1 cells relative to the control miRNA SNORD44. Student’s t-test was conducted (sh71 versus shNT): ****p < 0.0001 (**E**). Validation of significant DEGs identified by RNA-Seq through RT-PCR in CRC-SC#1 and CRC-SC#2 cells. Data represent the mean ± SD of at least 3 independent experiments (**F**).

To explore the cellular processes modulated by KDM1A in CRC, we performed a transcriptomic analysis of KDM1A-silenced CRC-SC#1 cells. By using DESeq2, a total of 89 genes were found to be significantly modulated upon KDM1A-silencing (|log2 FC| > 1 and p.adjusted < 0.1), consisting of 58 upregulated and 31 downregulated genes (Supplementary Table 2A). A heatmap showing unsupervised hierarchical clustering of the 89 deregulated genes (DEGs) is shown in Fig. 5A. The semantic plot obtained with ToppGene-identified biological processes (BP) associated with the 89 DEGs revealed the modulation of BP linked to cell differentiation, gland, and epithelium morphogenesis, along with the development of an exocrine system and transmembrane transport of organic acids/anions (Fig. 5B). Accordingly, g:Profiler output relative to Human Protein Atlas, showed 12 out of the 58 upregulated genes significantly associated with intestinal goblet, endocrine cells and/or enterocytes phenotype (Fig. 5C). Among downregulated DEGs, ToppGene-identified BPs disclosed the modulation of genes involved in retinoic acid biosynthesis, a well-established inducer of CSCs differentiation. Particularly, aldo-keto reductase family 1 member C1/C2 (AKR1C, AKR1C2), previously reported to inhibit retinoic acid production by converting retinaldehyde to retinol, were significantly downregulated [37]. Accordingly, analysis of single gene functions revealed that 3 out of 31 downregulated genes were tightly associated with stemness maintenance, particularly CD164 [38], epithelial splicing regulatory protein 1 (ESRP1) [39], and CD55 [40] (Fig. 5C). Gene Set Enrichment Analysis (GSEA) further confirmed our results (Supplementary Fig. 2).

Notably, 15 out of 89 DEGs were represented by lncRNA and pseudogenes, along with 1 small nuclear RNA (SNORA48) (Supplementary Fig. 3), and 3 of them were found among the top ten downregulated DEGs (log2 FC < −1.4), whereas 3 were in the top ten upregulated DEGs (log2 FC > 2.9) (Supplementary Table 2A). In particular, the pseudogene FABP5P7 was the most upregulated gene (log2 FC = 6.9; p.adjusted = 2.7E−03), while the lncRNA AC067931.1 was the most downregulated (log2 FC = −10.1; p.adjusted = 6.6E−09). The Ingenuity Pathway Analysis of DEGs predicted the modulation of several upstream regulators including the activation of miRNA-124-3p family (Supplementary Table 2B). This miRNA was further confirmed, together with miRNA-506-3p, as possible critical players in KDM1A-silenced cells by different miRNA prediction software (Fig. 5D). In KDM1A-silenced cells, although miRNA-124-3p did not display significant deregulation, miRNA-506-3p showed a 3-fold change increase (*p* < 1E−05) (Fig. 5E).

Validation of transcriptomic results was performed by investigating, through RT-qPCR, 11 DEGs involved in cell differentiation and targets of miRNA-124-3p and miRNA-506-3p. All tested DEGs revealed a good correlation between RT-qPCR and RNA seq in CRC-SC#1 cells (*ρ* = 0.92, *p* < 1E−4), and 7 DEGs were also validated in CRC-SC#2 cells (*ρ* = 0.97, *p* = 4E−4) (Fig. 5F).

### Proteomic analysis of KDM1A silenced cells unveils alterations of RNA metabolism, cytoskeleton remodeling, and mitochondrial function

**Fig. 6:**
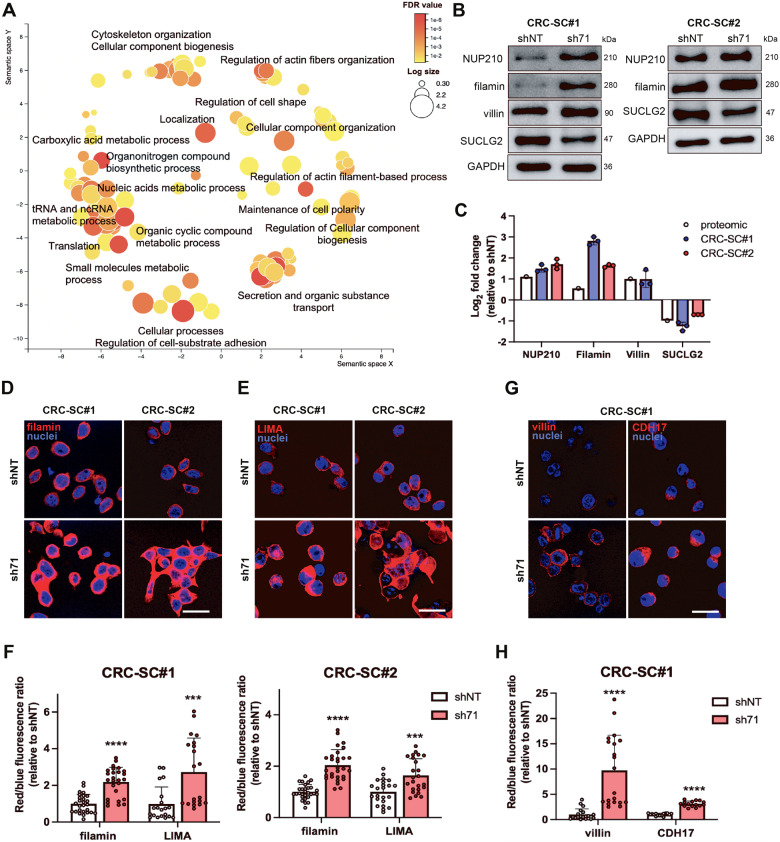
Proteomic analysis of CRC-SCs-silenced cells discloses alterations in RNA metabolism, mitochondrial function and cytoskeleton remodeling. Semantic plot of the enriched biological processes (**A**). Proteomic validation through western blot of NUP21, filamin, villin, and SUCLG2 representative images (**B**) and quantification in CRC-SCs (**C**) Data represent the mean ± SD of at least 3 independent experiments. Representative images of transduced CRC-SC#1 and CRC-SC#2 stained with topro (blue) and in red filamin (**D**), LIMA (**E**), villin, and CDH17 (**G**) (scale bar 25 μm), IF quantification (**F**, **H**). Data represent the mean of at least 3 independent experiments ± SD. Student’s t-test was conducted (sh71 versus shNT): ***p < 0.001; ****p < 0.0001.

To deeply investigate the cellular processes affected by KDM1A knockdown, we performed an unbiased proteomic analysis of CRC-SCs transduced with sh71 or shNT. Proteomic profiling revealed 136 differentially expressed proteins in KDMA1-silenced CRC-SCs (*p* < 0.05) (Supplementary Table 3). Among the 136 deregulated proteins, 90 were found to be upregulated whereas 46 were downmodulated. The protein-protein interaction network generated by the Search Tool for the Retrieval of Interacting Genes/Proteins (STRING) revealed protein-protein interactions significantly higher than expected by chance (*p* = 1.60E−16), indicative of a biological association between deregulated proteins upon KDM1A silencing (Supplementary Fig. 4). Gene ontology enrichment analysis disclosed significant modulation of several BP, including RNA processing, protein synthesis and transport, cell metabolism, actin cytoskeleton remodeling, and DNA repair (Fig. 6A, Supplementary Table 4).

Among proteins associated with cell metabolism, several mitochondrial proteins participating in the Krebs cycle and ATP production resulted downregulated, including Succinate-CoA Ligase GDP-Forming Subunit Beta (SUCLG2) and Succinate Dehydrogenase Complex Iron-Sulfur Subunit B (SDHB). However, experiments investigating mitochondrial dysfunction upon KDM1A silencing, including mitochondrial oxygen consumption rate and mitochondrial membrane polarization analysis, revealed regular mitochondrial functioning (Supplementary Fig. 5).

KDM1A silencing resulted in the enrichment of proteins involved in the regulation of actin filament remodeling as well as cell and anchoring junctions, including filamin (FLNA, FLNB), junction plakoglobin (JUP), coronin 1C (CORO1C), LIM domain and actin-binding protein 1 (LIMA1), Iq motif-containing GTPase activating protein (IQGAP1, IQGAP2), destrin (DSTN), capping actin protein of muscle z-line alpha subunit 2 (CAPZA2) (Supplementary Table 4).

The expression levels of 6 proteins, representative of cytoskeleton remodeling and metabolism, including the nuclear pore membrane glycoprotein 210 (NUP210), FLNA, LIMA1, and SUCLG2, found to be deregulated by proteomic analysis, were investigated, and confirmed, by western blot, in both CRC-SC#1 and CRC-SC#2 cells (Fig. 6B, C; Original Data File). The cytoskeletal proteins FLNA and LIMA1 were also validated by immunofluorescence analysis (Fig. 6D–F). Villin-1 (VIL1) was validated by western blot and confirmed by immunofluorescence analysis in CRC-SC#1 cells along with Cadherin 17 (CDH17), whose mRNA was also found to be upregulated by transcriptomic analysis (Fig. 6G, H).

Besides, several proteins deregulated upon KDM1A silencing participate in DNA repair, in particular, the bifunctional polynucleotide phosphatase/kinase (PNKP), the Ubiquitin-Like Modifier Activating Enzyme2 (UBA2), and KHDRBS1/Sam68 [41] were upregulated in KDM1A-silenced cells, whereas the TAR DNA Binding Protein (TARDBP), a member of the hnRNP family involved in nonhomologous DNA end joining and DNA repair, was downregulated [42] (Supplementary Table 4). The deregulation of those proteins further corroborates our data unveiling the involvement of KDM1A in DDR. An integrative analysis of proteomic and transcriptomic data can be found in Supplementary Fig. 6. Altogether our results reveal a crucial role of KDM1A in RNA metabolism, protein translation, cell metabolism, cytoskeletal organization, and DDR in CRC cells.

## Discussion

Epigenetic targeting is an area of growing interest in cancer treatment [43] and KDM1A overexpression is usually associated with poor prognosis, stemness maintenance, epithelial-to-mesenchymal transition (EMT), and escape of immune surveillance in a variety of cancers [17]. Though the role of KDM1A in the migration and invasion of differentiated CRC cells was previously reported [18], its complete characterization in terms of CRC-SCs remains poorly explored. Several efforts have been made to identify therapies for overcoming CSCs quiescence by inducing differentiation and/or cell death [44]. Here, we show that KDM1A silencing reduced cell viability and induced apoptosis in CRC-SCs derived from primary tumors. Coherently with the results of clonogenic assays, the transcriptomic analysis revealed a significant downregulation of genes associated with stemness maintenance in KDM1A-silenced CRC-SCs compared to control cells, while several genes, involved in intestinal cell differentiation, were overexpressed. Among these genes are CDH17, which regulates the morphological organization of the pancreatic ducts and the gastrointestinal tract [45], UGTA2A3, reported to be highly expressed in the proximal region of the intestinal epithelium [46] and HMGCS2, a rate-limiting ketogenic enzyme and marker of differentiated intestinal cells with a role in small intestinal and colon cells’ homeostasis maintenance [47]. Differentiation markers have also been identified by the proteomic approach. In particular, VIL1, participating in the organization of the microvillar cytoskeleton and the structure of the brush border [48], and LIMA1, a tumor suppressor [49] involved in the formation of cadherin-catenin complex’s interaction with the cytoskeleton and acting as a mechanosensitive regulator in the preservation of the apical-basal polarity [50].

The tissue-specific three-dimensional scaffolding provided by the cytoplasmic cytoskeleton has context-dependent organizing properties, particularly visible in the intestinal epithelium [51]. Thus, since stem cells' cytoskeleton is usually poorly developed, prominent modifications in their cytoskeleton are required with cell differentiation and lead to the inhibition of oncogenic features [52]. Notably, KDM1A-silenced CRC-SCs were significantly enriched in tumor suppressors involved in the regulation of actin filament depolymerization and polymerization processes as well as cell and anchoring junctions, including ADP-ribosylation factor-like protein 1 (ARL1), reported to be involved in cell polarity preservation [53], and galectin 1 and 4, associated with intestinal cells secretion and brush borders maintenance [54, 55].

The dynamic process of cytoskeleton remodeling is associated not only with cell differentiation but also with cell migration and invasion, a fundamental feature of EMT during carcinogenesis [56]. In line with our data of positive correlation between the expression of KDM1A and metastatic disease in CRC patients, our results aiming at investigating the invasive and migrative potential of CRC cells revealed a substantial decrease in both oncogenic features in KDM1A silenced cells, supporting previous evidence [57]. Likewise, proteomic analysis revealed a significant downmodulation of proteins involved in cell migration and interaction with the extracellular environment, including thromboxane A2 synthase1 (TBXAS1), usually associated with anchorage-independent growth and invasion [58]; integrin 6 (ITGA6) linked with CRC-SCs self-renewal maintenance [59]; the type I transmembrane protein chondroitin sulfate proteoglycan 4 (CSPG4) reported to potentiate glioblastoma cancer cells proliferation, migration, and adhesion [60]. Further studies should be addressed to fully investigate dynamic cytoskeleton remodeling and the cytoskeletal network, which governs the mechanoresponse in CRC-SCs upon KDM1A silencing.

Notably, several enzymes involved in mitochondrial metabolism were deregulated in KDM1A-silenced cells. Although the reduction of SUCLG2 in KDM1A-silenced cells could suggest succinyl-CoA accumulation, diminished production of succinate, and impaired progression of the tricarboxylic acid cycle, our experiments did not disclose mitochondrial impairment. Conversely, KDM1A silencing is likely to activate alternative pathways, namely ketone metabolism, and the GABA shunt [61], a pathway starting from glutamate being converted to GABA by glutamate decarboxylase [62]. GABA transaminates with α-ketoglutarate to form glutamate and succinate semialdehyde (SSA). SSA will get dehydrogenated by Aldehyde Dehydrogenase 5 Family Member A1(ALDH5A1) to yield succinate, and thus enter the Krebs cycle. SSA can also be converted to gamma-hydroxybutyrate (GHB) by Aldo-Keto Reductase Family 7 Member A2 (AKR7A2) [63]. Remarkably, both ALDH5A1 and AKR7A2 were significantly overexpressed in KDM1A-silenced cells, whereas high concentrations of endogenous GHB have been demonstrated in the rat and mouse gastrointestinal tract, including stomach, small intestine, and colon-rectum [64]. Remarkably, SUCLG2 was reported downregulated also in KDM1A-silenced glioblastoma cells [20] confirming a positive correlation between KDM1A and SUCLG2.

In an attempt to identify an upstream regulator for the identified DEGs upon KDM1A silencing, we observed the overexpression of miRNA-506-3p. Ai et al. demonstrated that miRNA-506-3p expression was lower in CRC tumor samples than in normal adjacent tissues, whereas miR-506-3p overexpression inhibited CRC cell proliferation, invasion, and migration leading to apoptosis [65]. Although previous publications demonstrated the regulation of miR-506-3p by lncRNA or circRNA in enhancing the expression of target genes [66], here we did not investigate further this issue. However, the regulatory network of miR-506-3p in cancer is of considerable relevance in tumor prevention and treatment, thus further studies are required to reach a more exhaustive understanding of its association with KDM1A.

Lastly, KDM1A has been reported to play a role in DDR [33], known to modulate stem cells’ differentiation [67]. KDM1A silencing induced H2AX phosphorylation and a strong reduction of 53BP1 DNA repair foci. These findings agree with the cell cycle progression impairment and the reduced expression of PLK1 and AURKA genes [68], observed in KDM1A-silenced cells. These findings are consistent with previous results demonstrating that KDM1A inhibition attenuates the PLK1 mitotic pathway in lung adenocarcinoma [30] and suggest a role for KDM1A in regulating cell cycle progression through the regulation of the PLK1-AURKA axis and DDR.

Despite numerous improvements, CRC remains a significant challenge, especially in advanced stages. It is now clear that most oncological-related deaths are caused by metastases [69] and that CSCs are intimately linked to metastases formation, as well as multidrug resistance and relapse [4]. Therefore, the combination of conventional cancer therapeutic such as surgery and radio-chemotherapy and specific targeted therapies, which consider both genetic and epigenetic alterations, appears to be a promising strategy in the fight against cancer. Herein, we underlined in vitro the important role of KDM1A in CRC cells survival and proliferation, as well as in promoting invasion and metastasis, studies should be addressed for the identification of biological processes modulated by KDM1A silencing that are the main ones responsible for the observed phenotype and for the in vivo validation of our findings. Also, we demonstrated the pivotal role of KDM1A in maintaining the clonogenic potential of CRC-SCs and their self-renewal capability. Our findings support the targeting of KDM1A in CRC therapy and pave the way for the development of an effective differentiation therapy overcoming the selection of resistant and aggressive clones triggered by conventional cytotoxic agents.

## Materials and methods

### Sample and data collection

The retrospective part of the study was carried out on 10% formalin-fixed material embedded in paraffin obtained from surgical samples of CRC. In the study, 41 cases of CRC representative of the investigated population were included, which had been treated surgically at the center of General Surgery of the Sant’Andrea of Vercelli Hospital in the period 2014 to 2019. The histological diagnoses were performed at the SCDU of Pathological Anatomy of the same center. None of the included patients had previously undergone neoadjuvant treatment and none of them had a concomitant or previous history of other cancers. The diagnoses were revised and reformulated according to the AJCC classification [28] and for each case, a paraffin embedding was selected for immunohistochemical studies. Data from the molecular analyses performed for predictive purposes were also collected (BRAF, K-RAS, etc.), in view of any pharmacological treatments. Clinical data were obtained from patients' medical records (gender, date of birth, diagnosis, surgery; grade and stage; margin status; adjuvant treatments performed; evidence of relapse/progression; last follow-up, and state of health).

### Immunohistochemistry

Immunohistochemical analysis was performed to determine KDM1A expression in tumor tissues, comparing it with the expression in non-neoplastic colon tissues. Evaluation of the results was performed using the Leica DM2500 microscope, equipped with a camera and microscopic picture projection on high-definition video to better specify the details of the positivity. Initially, 3-μm-thick sections were cut and de-refined as in use. Next, they were treated for 20–30 min in EDTA buffer in a microwave oven at 800 W for the unmasking of the antigen. Endogenous peroxidase was subsequently inhibited with hydrogen peroxide (H_2_O_2_) in 3% methanol for 30 min. At this point, the sections were incubated in the Banchark Ventana Ultra automatic immunostainer (Roche Diagnostics GmbH, Vienna, Austria) appropriately set. The primary monoclonal antibody used was an anti-KDM1A antibody (NB100-1762; Novus Biologicals, LLC, USA) diluted 1:1500. The positivity assessment was performed blinded by an expert eye. Positivity was assessed by giving a score to the intensity of nuclear staining (low or high) and was multiplied by the percentage of positive neoplastic cells evaluated in a semi-quantitative manner. Lastly, the cases in which the product was <80 were considered negative and positive for those with the product >80.

### Cell culture

Human differentiated CRC cells (HCT116, SW480) were purchased from the American Type Culture Collection (ATCC) and were cultured in Dulbecco’s modified Eagle medium (DMEM, Gibco, Waltham, MA, USA) supplemented with 10% fetal bovine serum (FBS, Euroclone, Milan, Italy) and 1% antibiotics–antimycotics (penicillin, streptomycin, and amphotericin; Sigma, Saint Louis, MO, USA).

Human CRC stem cell lines (CRC-SCs) CRC-SC#1, CRC-SC#2, CRC-SC#3, and CRC-SC#4 were kindly provided by Prof. Giorgio Stassi from the University of Palermo (Department of Surgical, Oncological, and Stomatological Sciences). Extraction and isolation were performed as previously reported [70]. Briefly, Cancer tissues were intensively washed with PBS supplemented with antibiotics and incubated overnight in DMEM/F12 (Gibco, Waltham, MA, USA) containing penicillin (500 U/ml), streptomycin (500 μg/ml), and amphotericin B (1.25 μg/ml) (Sigma-Aldrich, Saint Louis, MO, USA). Enzymatic digestion was performed using collagenase (1.5 mg/ml) (Gibco, Waltham, MA, USA) and hyaluronidase (20 μg/ml) (Sigma-Aldrich, Saint Louis, MO, USA) in PBS for 1 h. Then, the digests were used for the purification of CD133^+^ cells. Isolated CRC-SCs were cultured in suspension as colonospheres in stem cell medium (DMEM/F12, Gibco, Waltham, MA, USA) supplemented with EGF (10 μg/mL, PeproTech, Cranbury, NJ, USA) and FGF (20 μg/mL, PeproTech, Cranbury, NJ, USA), N2 and B27 (Gibco, Waltham, MA, USA), 1 mmol/L nicotinamide (Sigma-Aldrich, Saint Louis, MO, USA) and 1% antibiotics–antimycotics (penicillin, streptomycin, and amphotericin; Sigma-Aldrich, Saint Louis, MO, USA). All the cell lines were routinely tested for mycoplasma and cultured under a controlled temperature and atmosphere in a humidified incubator (37 °C, 5% CO_2_).

### Cell transduction

To generate stable KDM1A-knockdown cell lines we used MISSIONpLKO.1-puro Empty Vector Plasmid DNA (Sigma-Aldrich) harboring either the sequence targeting human LSD1 (TRCN0000046071; here sh71 or TRCN0000046068; here sh68) or a non-targeting short hairpin RNA (shRNA) (SHC002; here shNT). Differentiated CRC cells were plated and transduced with lentiviral particles in the presence of polybrene (6 µg/ml; Sigma-Aldrich, Saint Louis, MO, USA). After overnight incubation with lentiviral particles, cells were cultured in a complete culture medium supplemented with puromycin (1 µg/ml; Sigma-Aldrich, Saint Louis, MO, USA) allowing the selection of transduced cells only. 500,000 CRC-SCs were plated in an ultra-low 24-well plate and transduced with lentiviral particles in the presence of 6 µg/ml polybrene (Sigma-Aldrich, Saint Louis, MO, USA). Spinoculation protocol was performed as follows: the plate was centrifuged for 1 h at 37 °C, 1800 rpm. Subsequently, cells were resuspended and incubated for 1 h (37 °C, 5% CO_2_); lastly, medium containing viral particles was removed, and cells were resuspended in a fresh medium and incubated overnight (37 °C, 5% CO_2_). The following day, 1.5 µg/ml puromycin was added to each well for selection.

### Viability assay

For both differentiated and CRC-SCs, 1000 cells/well were plated in a final volume of 100 μL/well in a 96-well plate. Cells were plated 3 days after transduction or treated with different concentrations of drug and incubated for 72 h. The same concentration of vehicle was used as control. Subsequently, CellTiter-Glo (Promega, WI, United States) was added to each well following manufacture’s instruction. Luminescence was read at the spectrophotometer (Victor, PerkinElmer, Waltham, MA, USA).

### Western blot

Cells were washed in ice-cold PBS and solubilized with RIPA lysis buffer (25 mmol/L Hepes, pH 8; 132 mmol/L NaCl, 5 mmol/L EDTA, 1 mmol/L EGTA, 1 mmol/L ZnCl_2_, 50 mmol/L NaF, 1%, Nonidet P40, 10% Glycerol; all reagents were purchased from Sigma-Aldrich, Saint Louis, MO, USA) with protease inhibitors diluted 1:10 (AEBSF, aprotinin, bestatin, E-64, EDTA, leupeptin; Sigma-Aldrich, Saint Louis, MO, USA) and 265 nmol/L orthovanadate (Sigma-Aldrich, Saint Louis, MO, USA). The cellular lysates were kept in a wheel for 20 min at 4 °C and centrifugated at 12,500 × *g* for 15 min at 4 °C. Proteins pellet was collected and quantified with Pierce-BCA protein assay kit (Thermo Fisher Scientific, Waltham, MA, USA). Samples were denatured with 2% SDS (Sodium Dodecyl Sulfate; Sigma-Aldrich, Saint Louis, MO, USA), 150 mmol/L DTT (Dithiothreitol; Sigma-Aldrich, Saint Louis, MO, USA), and 0.01% bromophenol blue for 5’ at 95 °C and then loaded and run at 120 Volt for 2 h in polyacrylamide gels. Proteins were then transferred to a polyvinylidenedifluoride (PVDF) membrane (Amersham, Buckinghamshire, United Kingdom) with a semi-dry transfer (0.1 Ȧ for 90 min). The membrane was saturated with 3% BSA (Sigma-Aldrich, Saint Louis, MO, USA) for 1 h and then incubated with the specific primary antibody overnight. The membrane was then extensively washed with TBS-Tween (Sigma-Aldrich, Saint Louis, MO, USA) for 30 min and incubated with a secondary antibody (dilution 1:3000) in TBS 0.1% Tween-20 for 60 min. After three items of washing with TBS-Tween, the membranes were read with the ECL Western Lightning Chemiluminescence Reagent Plus (PerkinElmer Life Science, Waltham, MA, USA). Luminescence was acquired with ChemiDoc Touch (Bio-Rad, Hercules, CA, USA) and analyzed with ImageLab (Bio-Rad, Hercules, CA, USA). Primary antibodies were anti-KDM1A (#2139; Cell Signaling Technology, Danvers, MA, USA), anti-NUP210 (A301-795A-M; Thermo Fisher Scientific, Waltham, MA, USA), anti-Filamin (sc-17749; Santa Cruz Biotechnology, Dallas, TX, USA), anti-Villin (sc-58897; Santa Cruz Biotechnology, Dallas, TX, USA), anti-SUCLG2 (sc-390818; Santa Cruz Biotechnology, Dallas, TX, USA), and anti-GAPDH (D16H11; Cell Signaling Technology, Danvers, MA, USA).

### Apoptosis assay

Transduced HCT116 and CRC-SC#1 cells (60,000 cells/well) were seeded in 24-well plates and subsequently stained at different time points (4 and 6 days after transduction). Cells were stained with annexin/propidium iodide (Ax/PI) following the manufacturer’s instruction (AdipoGen, San Diego, CA, USA). Briefly, cells were incubated at room temperature for 15 min with Ax V-FITC diluted in Ax binding buffer (10 mmol/L HEPES/NaOH, pH 7.4, 140 mmol/L NaCl, 2.5 mmol/L CaCl_2_). Next, cells were washed and resuspended in Ax binding buffer, and PI was added before flow cytometry analysis (Attune Nxt, Flow Cytometer, Thermo Fisher Scientific, Waltham, MA, USA). Data analysis was performed with FlowJo, LLC software v10 (Ashland, OR, USA).

### Cell cycle assay

Cell cycle analysis was performed measuring by measuring DNA content with Propidium Iodide (PI) staining. HCT116 cells (60,000 cells/well) were transduced in 12-well plates, selected with puromycin, and starved for 18 h. 48 h after the addition of DMEM 10% FBS, cells were harvested, washed with ice-cold PBS, and fixed with 70% ethanol for 30 min. Fixed cells were then treated with RNAse A (20 µg/ml; Sigma-Aldrich, Saint Louis, MO, USA) for 45 min. Lastly, cells were stained with PI (50 µg/ml; Sigma-Aldrich, Saint Louis, MO, USA) and fluorescence was acquired with a cytofluorimeter (Attune Nxt, Flow Cytometer, Thermo Fisher Scientific, Waltham, MA, USA). Data were analyzed with FlowJo, LLC software v 10 (Ashland, OR, USA).

### Methylcellulose and colony formation assay

To assess the clonogenic potential of CRC-SCs, 200 cells were seeded in ultra-low 24-well plate in a final volume of 800 µL of methylcellulose (2 parts of MethoCult SF H4236, Stemcell Technologies, Inc., Vancouver, Canada, and 1 part of complete medium). Cells were incubated at 37 °C, 5% CO_2_ for 12 days. Subsequently, colonies of two wells were stained with 0.5 mg/ml MTT for quantification and area analysis, whereas colonies of the two remaining wells were disaggregated and resuspended in a complete fresh medium. Next, following the aforementioned protocol, a second plating of the obtained homogeneous cell suspension was performed. Lastly, pictures were acquired and analyzed with ImageJ Software v.1.52a (Ashland, OR, USA).

### Soft agar colony formation assay/anoikis assay

To allow an anchorage-independent growth, transduced HCT116 and SW480 cells (200 cell in 24-well plates) were seeded in noble agar and incubated at 37 °C with 5% CO_2_ for 20 days. DMEM 10% FBS was renewed every 4–5 days. Colonies were stained with 0.5 mg/ml MTT for quantification and area analysis with ImageJ Software v.1.52a (Ashland, OR, USA).

### Migration and invasion assays

Cells were plated in specific 2-sided inserts (GmbH, Martinsried, Germany), 30,000 cells on each side. The inserts were then removed, and pictures were taken at different time points following drug treatment or plating of transduced cells. The percentage of wound area was assessed by ImageJ software v.1.52a (Ashland, OR, USA). To perform the invasion assay, transduced cells were seeded in the upper side of a Transwell Permeable Support (Corning, NY, USA) coated with Geltrex matrix (Thermo Fisher Scientific, Waltham, MA, USA). The number of invading cells was assessed by DiffQuick (Mendion Diagnostic, Fribourg, Switzerland) staining after 24 or 48 h.

### Confocal microscopy

Transduced cells were plated on round cover glasses and incubated at 37 °C and 5% CO_2_ in DMEM 10% FBS and, once attached, were pretreated with oxaliplatin (10 µmol/L) for 8 h. Oxaliplatin was then removed, and the cells were stained and mounted on microscope slides after 8 and 24 h after treatment removal. Cells were washed two times with PBS, fixed with paraformaldehyde 3%, and then washed with PBS. Subsequently, cells were permeabilized using HEPES-TRITON X-100 0.5% at 4 °C for 5 min, then washed three times with PBS containing 10 mM HEPES and 0.2% BSA and incubated with PBS-HEPES and 2% BSA at 37 °C for 15 min. Afterward, cells were stained with the primary antibody (1:100): anti-53BP1 (A300-272A-M; Thermo Fisher Scientific, Waltham, MA, USA), anti-p-H2A.X (14-9865-80; Thermo Fisher Scientific, Waltham, MA, USA). Similarly, for other analysis shKDM1A and control, cells were stained with: anti-Filamin (sc-17749; Santa Cruz Biotechnology, Dallas, TX, USA), anti-Villin (sc-58897; Santa Cruz Biotechnology, Dallas, TX, USA), anti-LIMA (sc-136399; Santa Cruz Biotechnology, Dallas, TX, USA), anti-CDH17 (MA5-29135; Thermo Fisher Scientific, Waltham, MA, USA), for 2 h at room temperature, washed and incubated with PBS-HEPES and 2% BSA for 15 min. Next, cells were stained with the secondary antibody (conjugated with Alexa Fluor 546; Invitrogen, Waltham, MA, USA) and TOPRO3 (Invitrogen, Waltham, MA, USA), washed three times with PBS-HEPES and 0.2% BSA, and mounted on microscope slides using the mounting media Mowiol (20% Mowiol 4–88, 2.5% DABCO in PBS, pH 7.4). Images and foci were detected by confocal microscopy and the number of foci was assessed through ImageJ software (Ashland, OR, USA). Images were acquired and subsequently analyzed through ImageJ software (Ashland, OR, USA).

### Proteomic analysis

The samples were subjected to denaturation with TFE, to reduction with DTT 200 mM, to alkylation with IAM 200 mM, and to complete protein digestion with 2 μg of Trypsin/Lys-C (Promega, Madison, WI, USA). The peptide digests were desalted on the Discovery® DSC-18 solid phase extraction (SPE) 96-well plate (25 mg/well) (Sigma-Aldrich Inc., St. Louis, MO, USA). The SPE plate was preconditioned with 1 mL of acetonitrile and 2 mL of water. After loading the sample, the SPE was washed with 1 mL of water. The adsorbed proteins were eluted with 800 μL of acetonitrile and water (80:20). Proteins were analyzed with the micro-LC Eksigent Technologies (Eksigent, Dublin, USA) system that included a micro LC200 Eksigent pump with flow module 5–50 μL, interfaced with a 5600+ TripleTOF system (AB Sciex, Concord, Canada) equipped with DuoSpray Ion Source and CDS (Calibrant Delivery System). The stationary phase was a Halo C18 column (0.5 × 100 mm, 2.7 μm; Eksigent Technologies Dublin, USA). The mobile phase was a mixture of 0.1% (v/v) formic acid in water (A) and 0.1% (v/v) formic acid in acetonitrile (B), eluting at a flow rate of 15.0 μL min^−1^ with increasing concentration of solvent B from 2% to 40% in 30 min. The injection volume was 4.0 μL and the oven temperature was set at 40 °C. For identification purposes the samples were subjected to data-dependent acquisition (DDA): mass spectrometer analysis was performed using a mass range of 100–1500 Da (TOF scan with an accumulation time of 0.25 s), followed by an MS/MS product ion scan from 200 to 1250 Da (accumulation time of 5.0 ms) with the abundance threshold set at 30 cps (35 candidate ions can be monitored during every cycle). The ion source parameters in electrospray positive mode were set as follows: curtain gas (N2) at 25 psig, nebulizer gas GAS1 at 25 psig, and GAS2 at 20 psig, ion spray voltage floating (ISVF) at 5000 V, source temperature at 450 °C and declustering potential at 25 V. For the label-free quantification process the samples were subjected to cyclical data-independent analysis (DIA) of the mass spectra, using a 25-Da window: the mass spectrometer was operated so that a 50-ms survey scan (TOF-MS) was performed and subsequent MS/MS experiments were performed on all precursors. These MS/MS experiments were carried out in a cyclical manner using an accumulation time of 40 ms per 25-Da swath (36 swaths in total) for a total cycle time of 1.5408 s. The ions were fragmented for each MS/MS experiment in the collision cell using the rolling collision energy. The MS data were acquired with Analyst TF 1.7 (AB SCIEX, Concord, Canada). A DDA and DIA acquisitions were performed. Peptides (and proteins) were identified using DDA followed by database search while the quantification was obtained by integrating the area under the chromatographic peak for each ion fragment of identified peptides by using the DIA file. The DDA files were searched using Protein Pilot software v. 4.2 (SCIEX, Concord, Canada) and Mascot v. 2.4 (Matrix Science Inc., Boston, USA). Trypsin as digestion enzyme was specified for both software. For Mascot we used 2 missed cleavages, set the instrument to ESI-QUAD-TOF and specified the following modifications for the assay: carbamidomethyl cysteine as fixed modification and oxidized methionine as variable modification. An assay tolerance of 50 ppm was specified for peptide mass tolerance, and 0.1 Da for MS/MS tolerance. The peptide charges to be detected were set to 2+, 3+, and 4+, and the assay was set on monoisotopic mass. The UniProt Swiss-Prot reviewed database containing human proteins (version 2015.07.07, containing 42,131 sequence entries) was used and a target-decoy database search was performed. False Discovery Rate was fixed at 1%. Quantification was performed by integrating the extracted ion chromatogram of all the unique ions for a given peptide. SwathXtend was employed to build an integrated assay library with the DDA acquisitions, using a protein FDR threshold of 1%. Quantification was carried out with PeakView 2.0 and MarkerView 1.2 (ABSCIEX, Concord, Canada). The six peptides per protein with the highest MS1 intensity and six transitions per peptide were extracted from the SWATH files. Shared peptides were excluded as well as peptides with modifications. Peptides with FDR lower than 1.0% were exported in MarkerView for the t-test.

### Transcriptomic analysis

Extraction of total RNA from CRC-SC#1 cells was performed using the miRNeasy® Mini Kit (Cat No./ID: 217004 QIAGEN, Venlo, The Netherlands), according to the manufacturer’s instructions.

RNA concentration and integrity were checked on a NanoDrop 2000 spectrophotometer (Thermo Fisher Scientific, Waltham, MA, USA) and on a Bioanalyzer 2000 (Agilent, Santa Clara, CA, USA), respectively. Specifically, RNA 6000 nano assay was employed. 500 ng of high-quality RNA (RIN > 9) was used to prepare RNA-libraries using TruSeq Stranded Total RNA Kit (Illumina, San Diego, CA, USA) according to manufacturer’s instructions. Library quality was checked with a Bioanalyzer DNA High Sensitivity assay (Agilent, Santa Clara, CA, USA). Libraries were multiplexed, clustered, and sequenced on an Illumina NovaSeq 6000.

Fastq files were processed with Cutadapt [71] to trim TruSeq adapters. Two biological replicates were analyzed for a total of 4 samples. The STAR program [72] was used to align reads to the hg38 human reference genome. RSEM computational pipeline [73] was used to measure the expression level of each human gene annotated in the Ensembl v100 database. Only genes with a TPM > 1 in at least one sample were considered expressed and underwent further analysis. Differentially expressed genes (DEGs) were calculated using DESeq2 [74] with the following parameters: |log2FC| > 1 and p.adj < 0.1. Heatmaps showing unsupervised hierarchical clustering of DEGs were produced using the “pheatmap” package of R. Characterization of enriched GO Terms in modulated genes and microRNA interactions, filtered by “TargetScan” interactions, were analyzed using ToppGene Suite software [75], using the default parameter of FDR < 0.05 to identify statistically significant enriched terms. The characterization of upstream regulators of modulated genes was computed using Ingenuity Pathway Analysis (IPA) (Qiagen Inc., Venlo, The Netherlands). Semantic plots were produced starting from the GO Terms predicted by ToppGene using Revigo software [76], setting the analysis to *Homo sapiens* and using default parameters. Upregulated DEGs were analyzed with g:Profile suite [77] results were used to create Fig. 5C with Biorender.

### RNA extraction and real-time PCR

To perform RNA extraction, 300,000 cells/well were plated for shNT and for sh71. RNA was extracted using miRNeasy® Mini Kit (Cat No./ID: 217004 QIAGEN, Venlo, The Netherlands), according to the manufacturer’s instructions or the phenol/chloroform method (RNAzol, Sigma-Aldrich, Saint Louis, MO, USA) and isopropanol precipitation, following the manufacturer’s instructions. RNA samples were quantified using a NanoDrop 2000 (Thermo Fisher Scientific, Waltham, MA, USA) and then reverse-transcribed into cDNA using recombinant Moloney murine leukemia virus reverse transcriptase (MultiScribe Reverse Transcriptase, Bio-Rad, Hercules, CA, USA) and the iScript cDNA Synthesis Kit (Bio-Rad, Hercules, CA, USA). The genes analyzed by real-time PCR using the SsoAdvanced Universal SYBR Green Supermix Kit (Bio-Rad) are reported in Table 1. HPRT was used as the control gene. Relative quantification was determined using the ΔΔCt algorithm.

**Table 1:** Oligo sequences (5′–3′) for the genes investigated.

Target gene	Forward	Reverse
BUB1	TCATTCATGGAGACATTAAAC	CTGAGCATCTCAACACACTG
PLK1	GGCAACCTTTTCCTGAATG	AATGGACCACACATCCACCT
AURKA	CAGGCAACCAGTGTACCTCA	GCCAGTTCCTCCTCAGGATT
CDH17	GCCAATCCTCCTGCTGTG	GCAACCTGGAGATTGTGAGT
UGT2A3	ATGAGGTCTGACAAGTCAGCTT	TCAAAATCCCAATATGTTCG
CCKAR	GCGATTTGCAAACCCTTACAG	CACCTTCAAAGCATGGGATTTT
TUBA1A	GGCAGTGTTTGTAGACTTGGAACCC	TGTGATAAGTTGCTCAGGGTGGAAG
HMGCS2	GGTTTCTGCTTG CTCCTCTG	TATGATTCACG GGG AGAAGC
SUCLG2	GGAGGAAGAGGAAAAGGTGTC	TTCAGCAACCATCACCTTGTT
ESRP1	CAATATTGCCAAGGGAGGTG	GTCCCCATGTGATGTTTGTG
CD164	GGCACCAGAAACCTGTGAAG	TGTCGTGTTCCCCACTTGAC
AKR1C1	ATTTGCCAGCCAGGCTAGTG	AGAATCAATATGGCGGAAGCC
SLC50A1	TCACCCTTGGCATGTTCTCC	ACTTCCGTGGTGAGAAAGGG
KDM1A	AGACGACAGTTCTGGAGGGTA	TCTTGAGAAGTCATCCGGTCA
HPRT	AAGGACCCCACGAAGTGTTG	GGCTTTGTATTTTGCTTTTCC

### miRNA extraction and real-time PCR

For miRNA extraction, 500,000 cells/condition were processed with Maxwell® RSC miRNA Tissue KIT (code: AS1460; Promega, Madison, WI, USA) according to the manufacturer’s instructions. RNA samples were quantified using a NanoDrop 2000 and then reverse-transcribed into cDNA, after poly-A tailing, with qScrip® microRNA cDNA Synthesis Kit (Quantabio, Qiagen, Beverly, MA) following the provider’s protocol. Real-time SYBR Green qRT-PCR was performed by using the PerfeCta SYBR® Green SuperMix family from the qScript microRNA Quantification System (Quantabio). Primers for miRNA investigation are reported in Table 2. SNORD44 was used as control. Relative quantification was determined using the ΔΔCt algorithm.

**Table 2:** Oligo sequences (5′–3′) for miRNAs investigated.

Target gene	Forward	Universal reverse primer
SNORD44	GCATAGACCTGAATGGCGGTA	GCATAGACCTGAATGGCGGTA
miRNA 124-3p (hsa-miR-124-3p)	TAAGGCACGCGGTGAATGC	GCATAGACCTGAATGGCGGTA
miRNA 506-3p (hsa-miR-506-3p)	CGTAAGGCACCCTTCTGAGT	GCATAGACCTGAATGGCGGTA

### Intact cell respiration using high-resolution respirometry

We determined cellular respiration using high-resolution respirometry using the substrate, uncoupler, inhibitor, and titration (SUIT) protocols as previously reported [78]. Briefly, Transduced CRC-SC#1 were centrifuged at 300 × *g* for 5 min, resuspended in mitochondrial respiration medium MiR05 (0.5 mM EGTA, 3.0 mM MgCl_2_·6H_2_O, 60 mM potassium lactobionate, 20 mmol/L taurine, 10 mmol/L KH_2_PO_4_, 20 mmol/L Hepes, 110 mmol/L sucrose, 1 g/L bovine serum albumin, pH 7.1) and transferred to an Oxygraph-2 K high-resolution respirometer (Oroboros Instruments, Innsbruck, Austria). shNT and sh71 samples were assessed simultaneously. After initial stabilization of O_2_ flux, pyruvate (5 mmol/L) was used to sustain TCA-linked respiration. ATP synthetase inhibitor, oligomycin (O), was added at 5 nmol/L final concentration, and oxygen consumption was quantified to determine the oligomycin-sensitive and -insensitive respiration. Protonophore (H^+^ ionophore) and uncoupler of oxidative phosphorylation, FCCP (U) were then added at 0.5 μmol/L increments to achieve maximum respiration to quantify maximum respiratory capacity. This was followed by rotenone (Rot) 500 nmol/L final concentration, to inhibit complex I of the electron transport chain (ETC), and then 2.5 μmol/L antimycin A (Aa), which inhibits complex III, was added to determine the non-mitochondrial respiration (ROX). Oxygen consumption rates were calculated using accompanying software (DatLab7, Oroboros). Rates of O_2_ consumption (flux) were normalized to protein total content.

### Mitochondrial assays

To evaluate mitochondrial membrane depolarization, transduced CRC-SC#1 cells were plated at a concentration of 30,000 cells/well in a 48-well plate pre-coated with Geltrex (Thermo Fisher Scientific, Waltham, MA, USA). Cells were stained with 10 μg/ml JC-1 dye (Adipogen, San Diego, CA, USA) in PBS for 30 min in the dark at 37 °C. FCCP (Cayman Chemicals, Ann Arbor, MI, USA) was added for 15 min at the end of the staining as a positive control. Signals were acquired with a fluorescence microscope (FLoid Cell Imaging Station, Life Technology) and images were analyzed by ImageJ software to determine the red/green fluorescence ratio. Mitochondrial morphology was assessed through Mitotracker CMX-Red (Invitrogen, Waltham, MA, USA). CRC-SC#1 cells were seeded at a concentration of 100,000 cells/well in a 24-well plate pre-coated with Geltrex. Subsequently, cells were stained with 10 nmol/L Mitotracker for 30 min at 37 °C. Fluorescence was visualized by DM5500B fluorescence microscope (Leica, Wetzlar, Germany), and analyzed using ImageJ software v 1.52a (Ashland, OR, USA).

### Statistical analysis

For statistical analysis of KDM1A expression in CRC patients’ frequencies were used to express categorical data. The correlation between KDM1A expression and the tumor stage of the patients was evaluated using the chi-squared test. Also, the progression-free survival (PFS) and overall survival (OS) of KDM1A positive and KDM1A negative patients were compared using Kaplan–Meier (KM) survival curves and log-rank analysis. Lastly, Cox regression analysis was conducted and stratified by patients' sex, age, tumor stage, and KDM1A expression, hazard ratio (HR) and confidence interval (95% IC) were calculated (all the described patient and disease characteristics have been included in the multivariable regression model). Statistical analyses were performed through R (survival and visualization package) version 2022.12.0 + 353. A *p*-value of <0.05 was considered statistically significant.

Experimental data were analyzed and presented as mean ± standard deviation (SD) or standard error of the mean (SEM) in all figures. Pairs of data groups were analyzed using paired and unpaired two-tailed Student’s t-test. Statistical significance was determined using Prism 9 (GraphPad Software, LCC, San Diego, California). Each experiment was repeated at least three times. Results with a *p*-value lower than 0.05 were considered significant; ****p < 0.0001, ***p < 0.001, **p < 0.01, *p < 0.05 compared to controls.

## Supplementary information

Suppl Fig 1

Suppl Fig 2

Suppl Fig 3

Suppl Fig 4

Suppl Fig 5

Suppl. Fig 6

Supplementary Figure Legends

Supplementary Table 1

Supplementary Table 2

Supplementary Table 3

Supplementary Table 4

Original Data File

## Data Availability

Raw reads and processed sequencing data were deposited in the NCBI Gene Expression Omnibus and are publicly available under accession number GSE216513. Proteomic data are available via ProteomeXchange with the identifier PXD037259.
