# Supplementary figures and images for: Targeting lysine-specific demethylase 1 (KDM1A/LSD1) impairs colorectal cancer tumorigenesis by affecting cancer cells stemness, motility, and differentiation
Source: Cell Death Discov. 2023 Jun 29;9:201. doi: 10.1038/s41420-023-01502-1 (PMC10310788; doi:10.1038/s41420-023-01502-1)

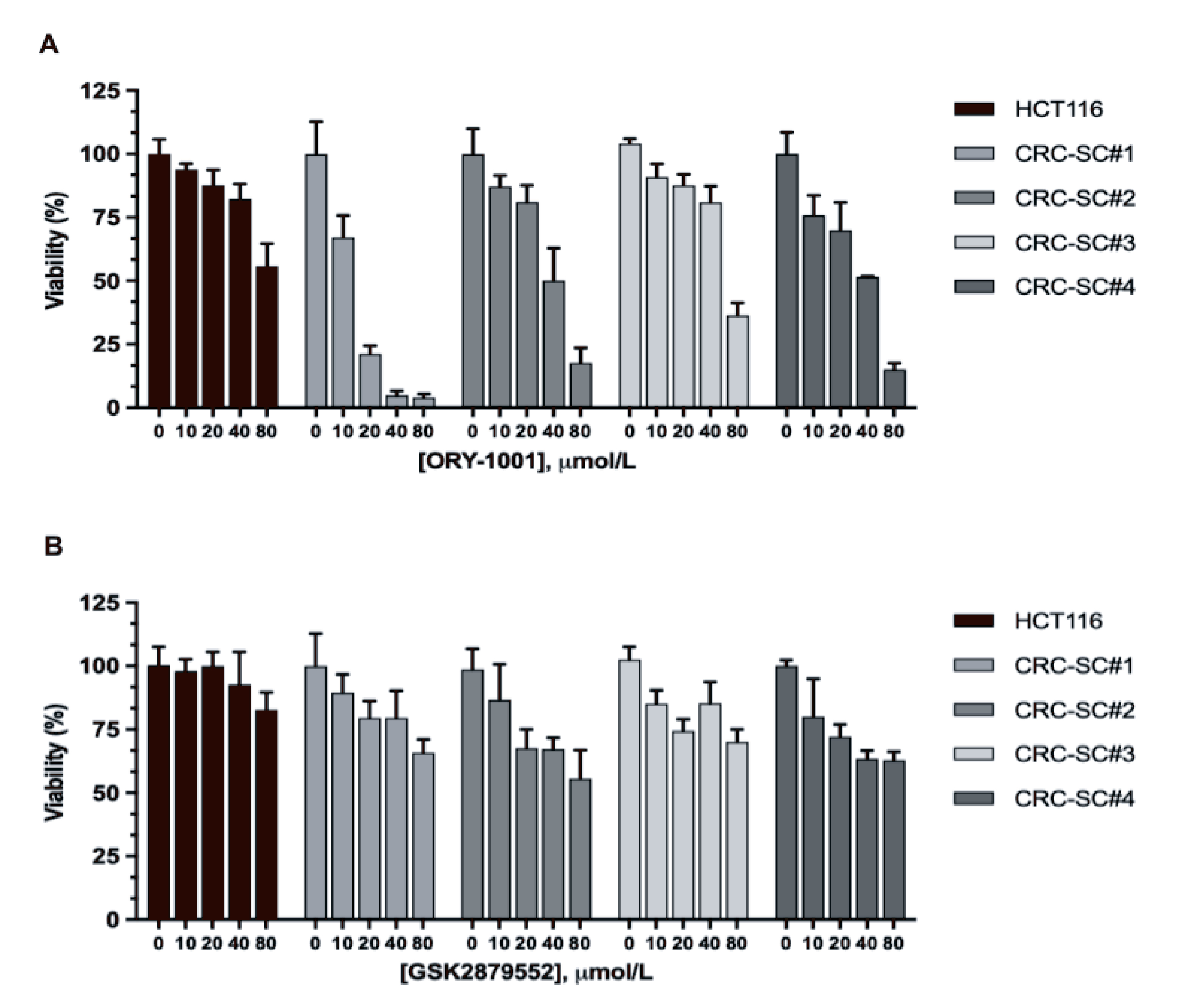

Supplement: Supplementary file 1 — Suppl Fig 1 [file 41420_2023_1502_MOESM1_ESM.jpg]

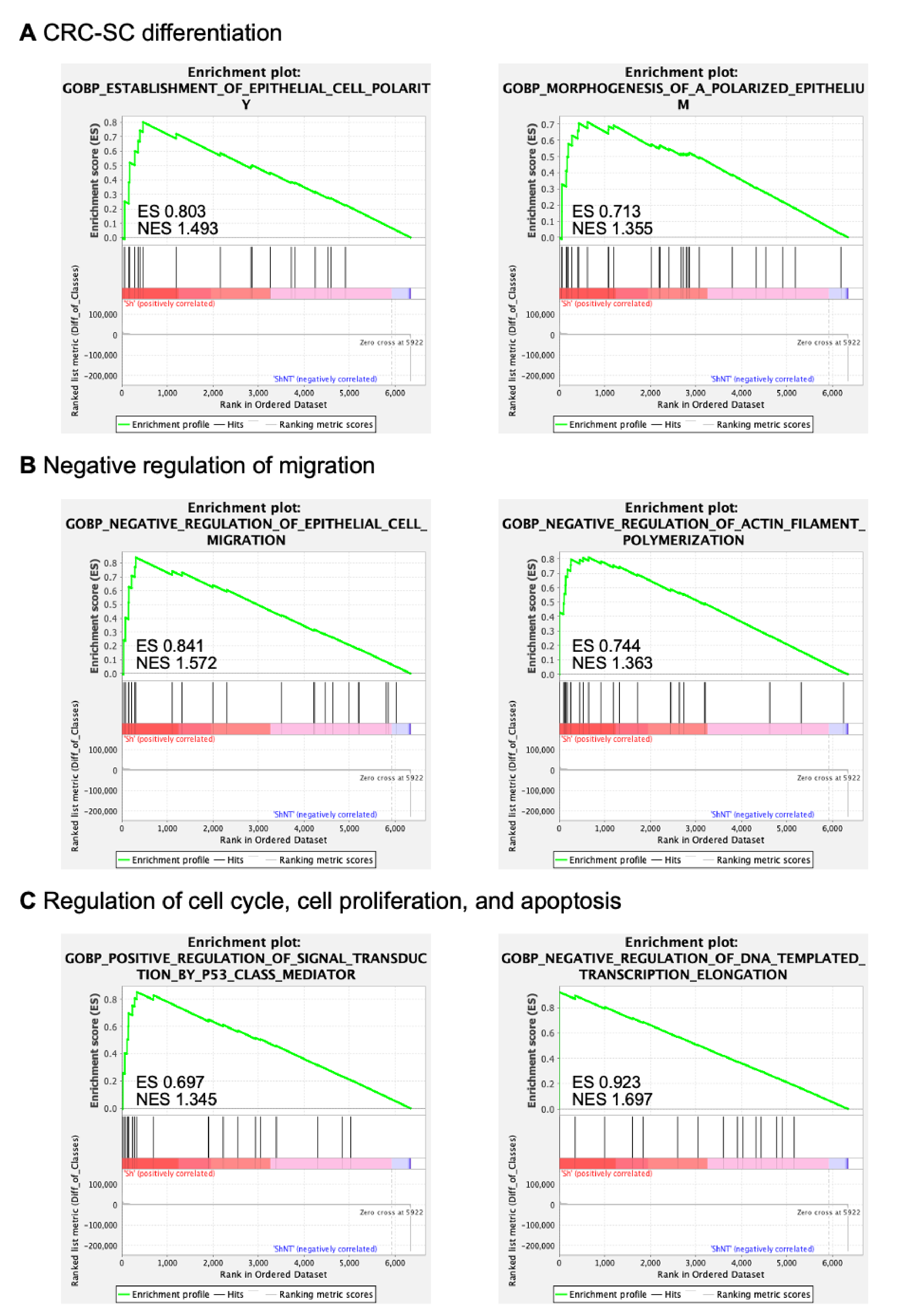

Supplement: Supplementary file 2 — Suppl Fig 2 [file 41420_2023_1502_MOESM2_ESM.jpg]

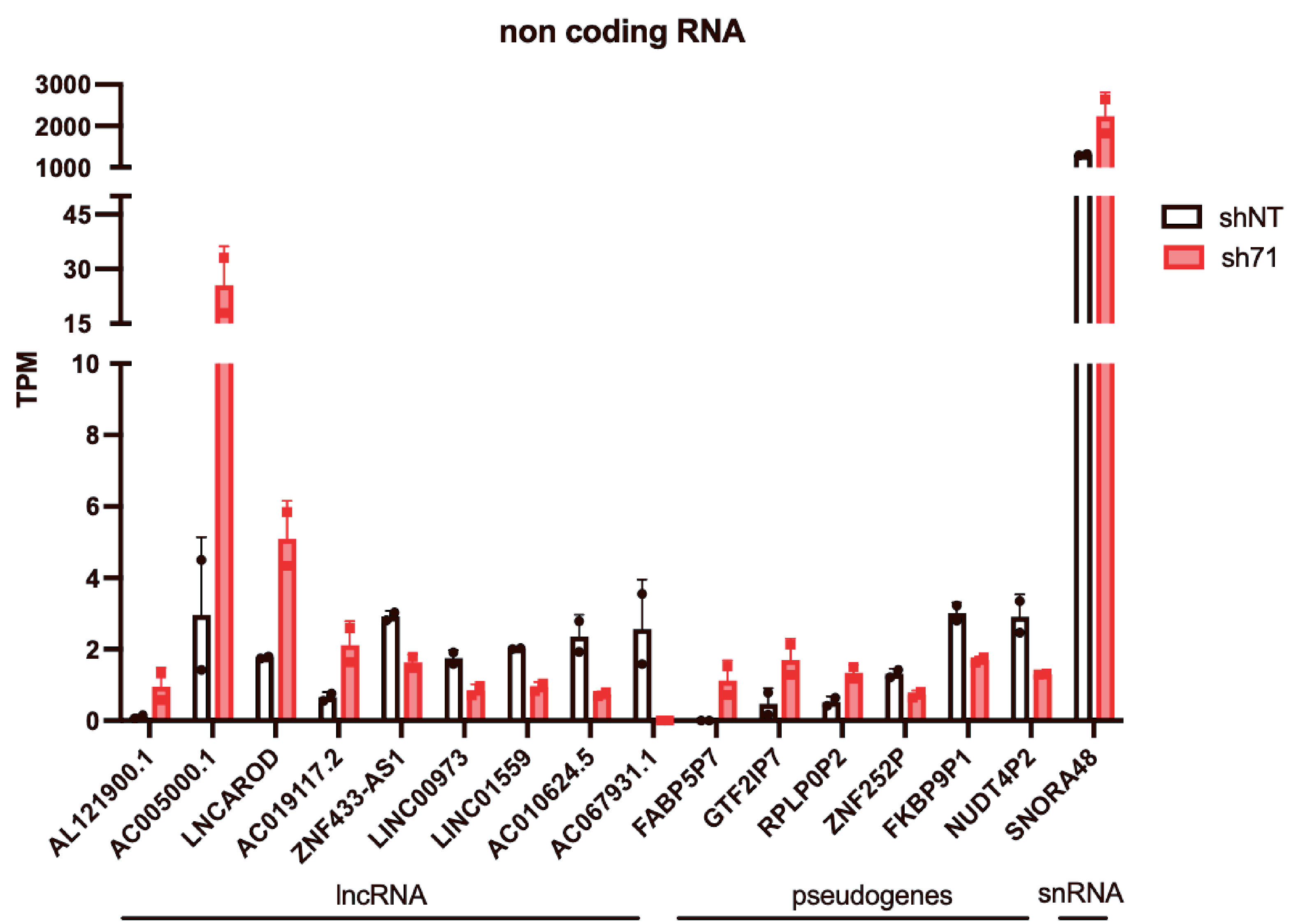

Supplement: Supplementary file 3 — Suppl Fig 3 [file 41420_2023_1502_MOESM3_ESM.jpg]

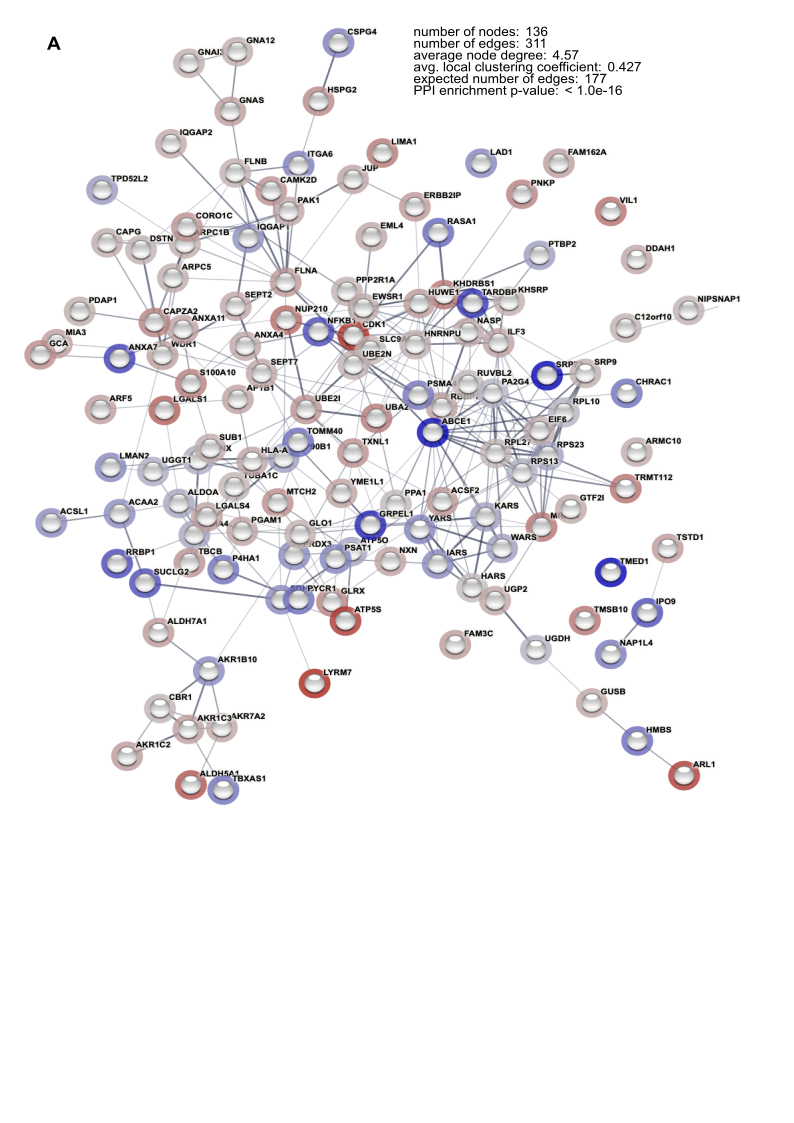

Supplement: Supplementary file 4 — Suppl Fig 4 [file 41420_2023_1502_MOESM4_ESM.jpg]

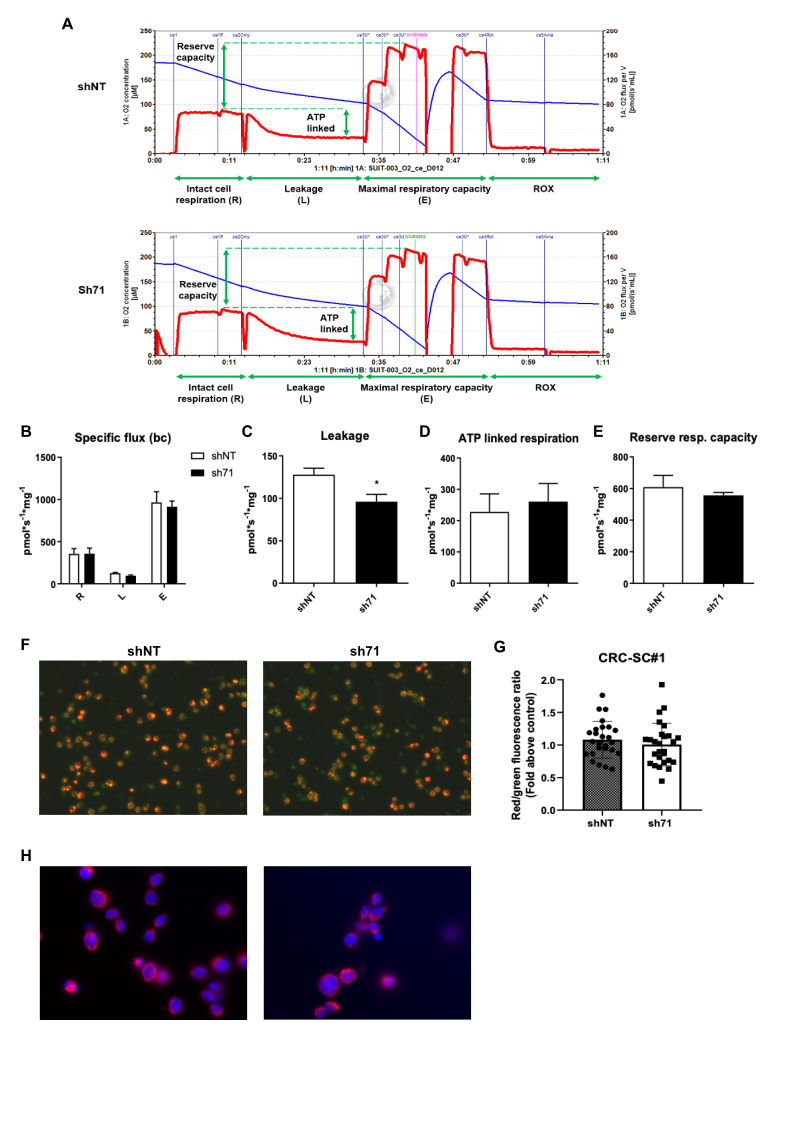

Supplement: Supplementary file 5 — Suppl Fig 5 [file 41420_2023_1502_MOESM5_ESM.jpg]

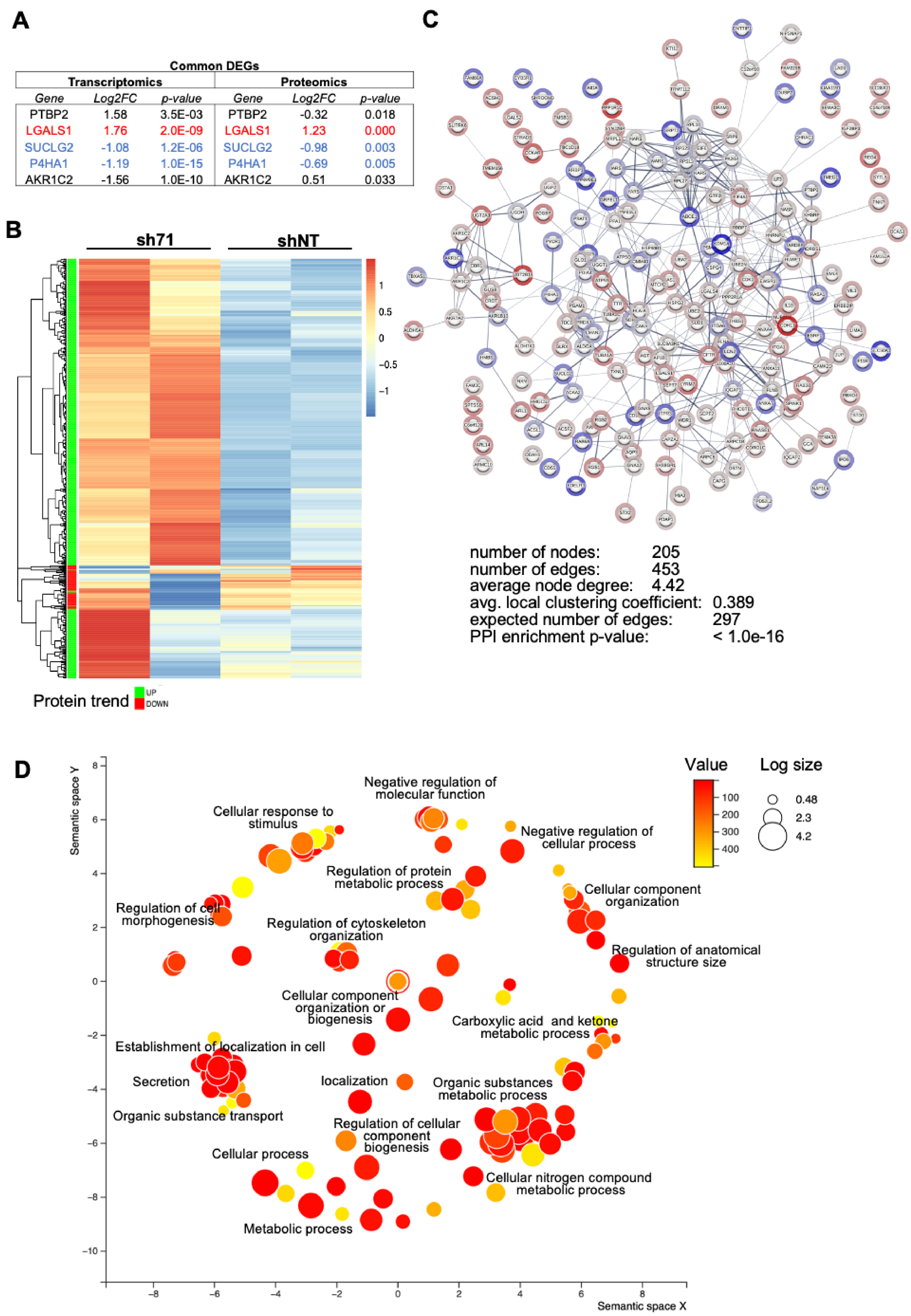

Supplement: Supplementary file 6 — Suppl. Fig 6 [file 41420_2023_1502_MOESM6_ESM.jpg]

Membranes of Fig. 2A

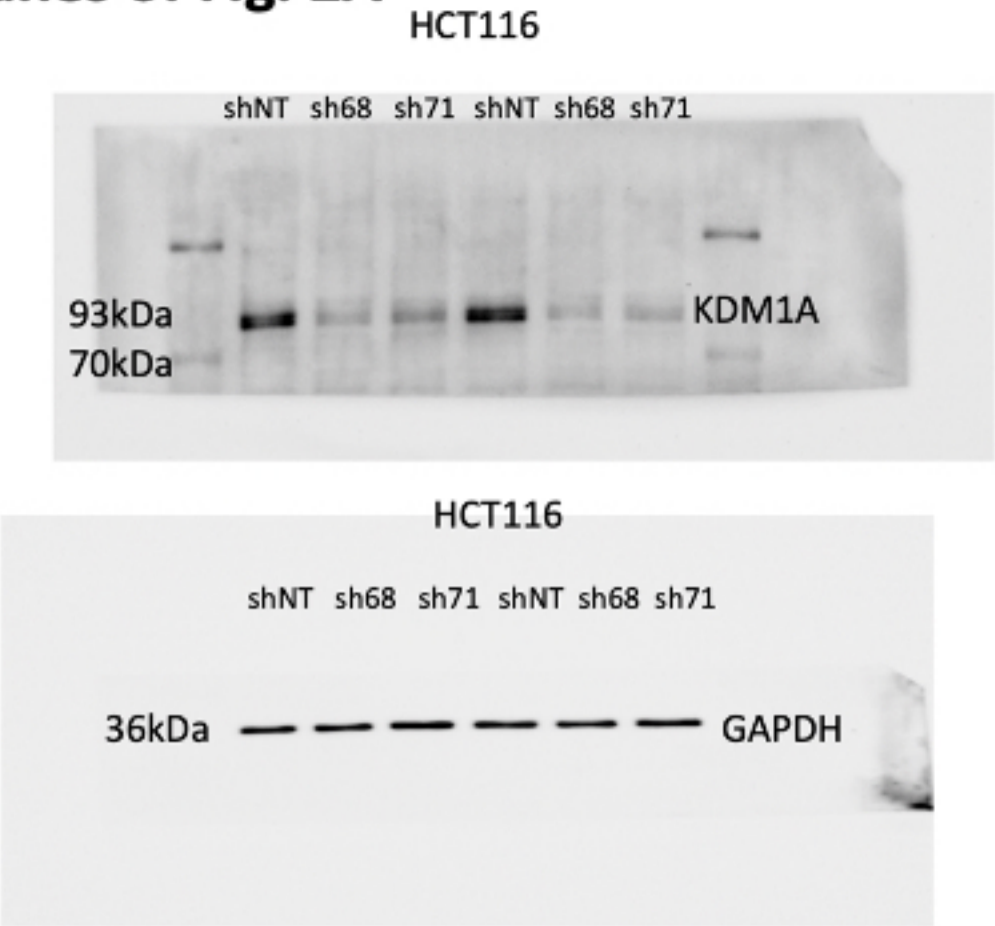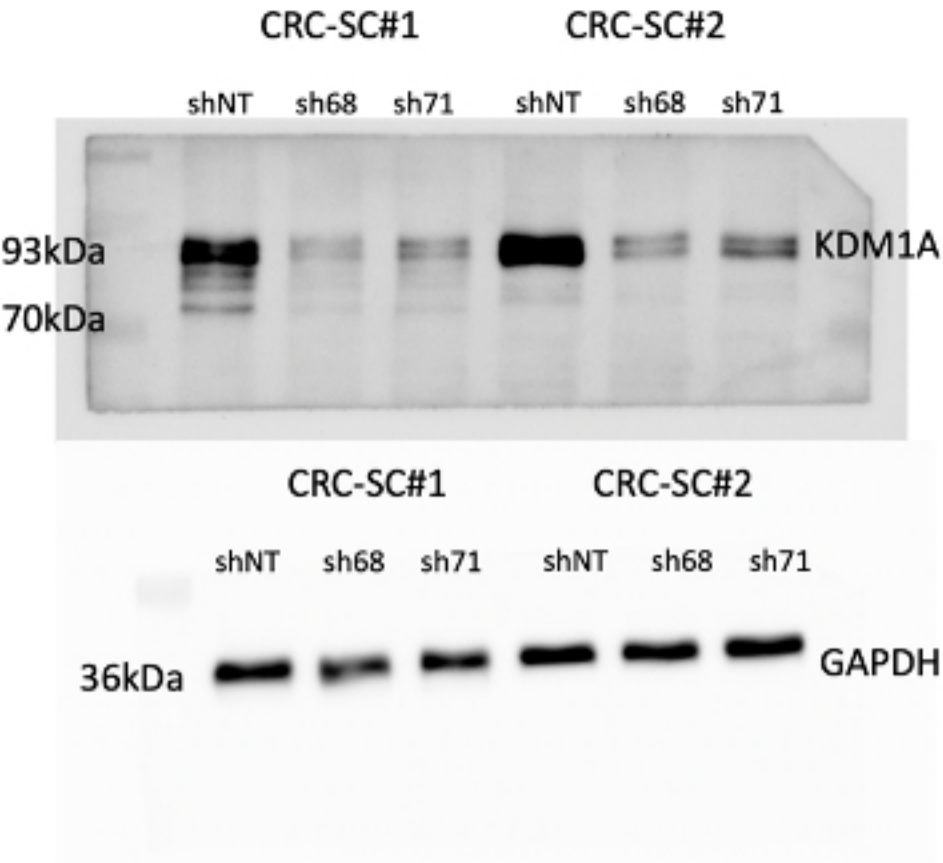

Membranes of Fig. 6B

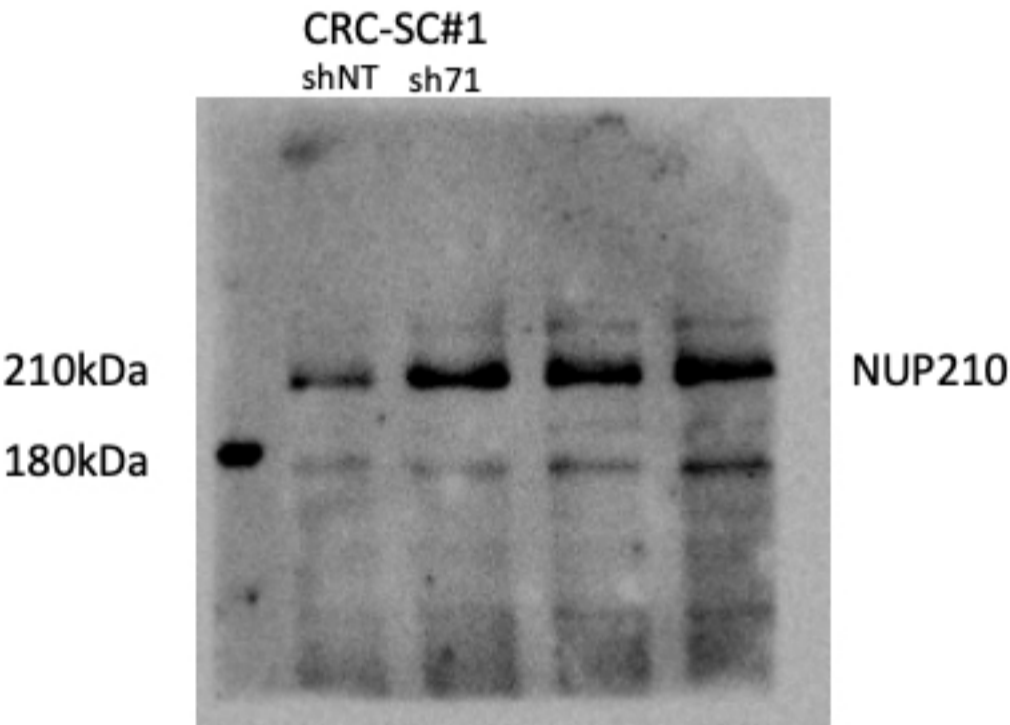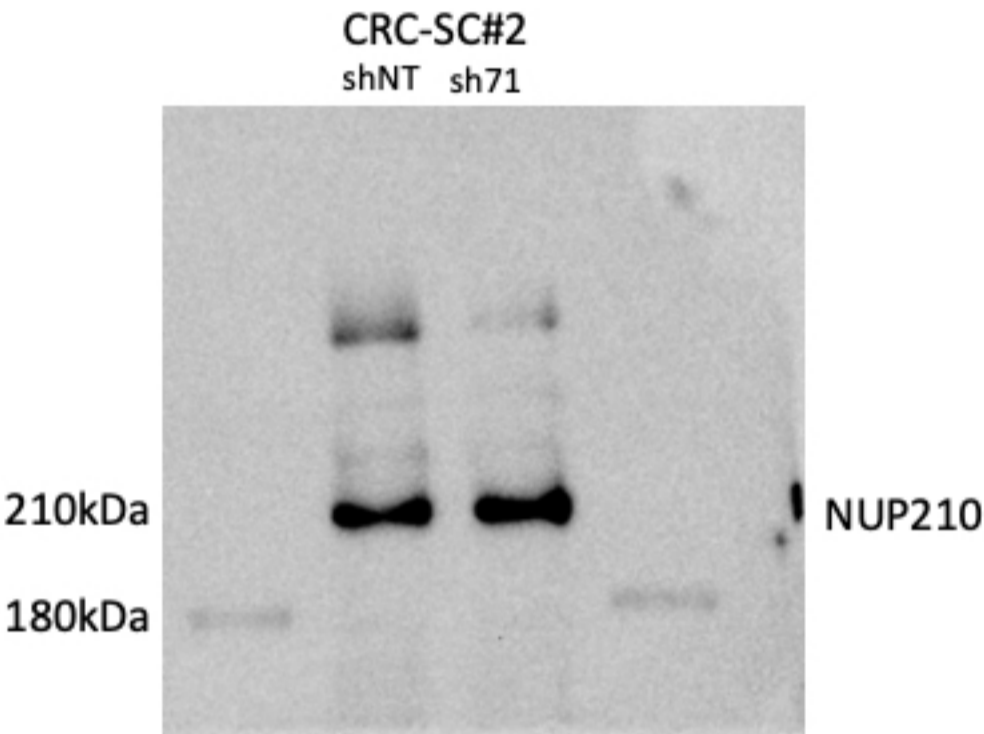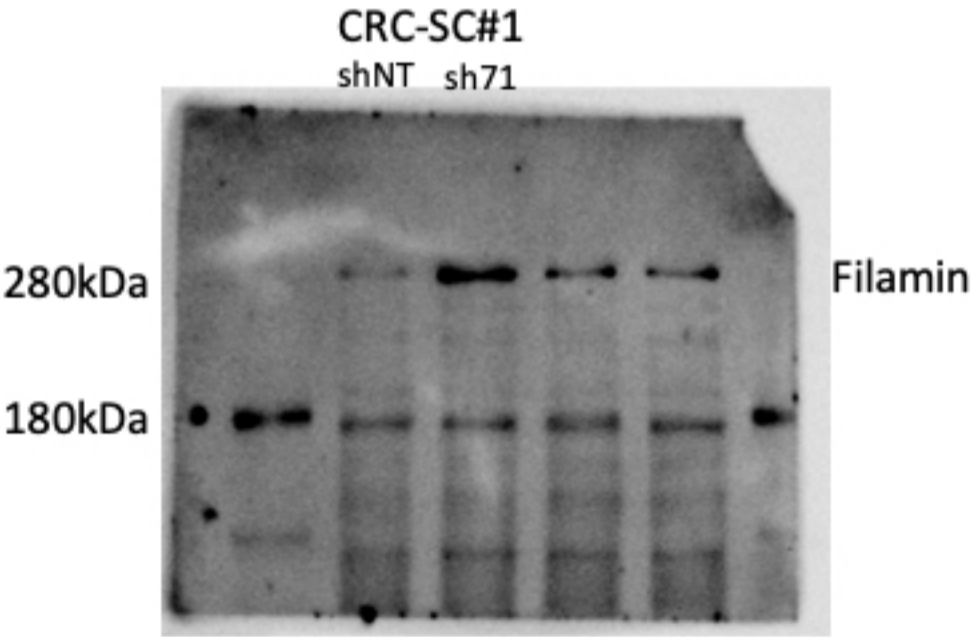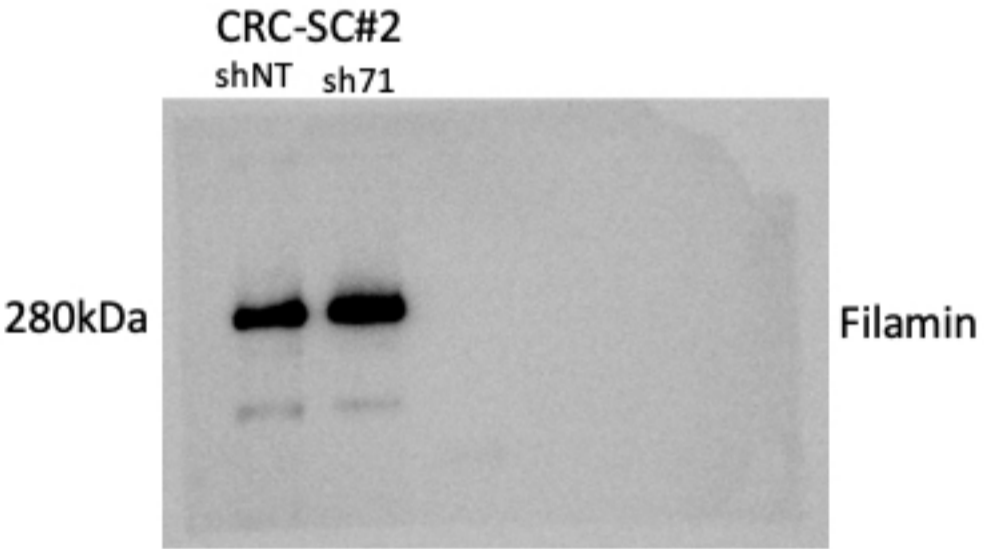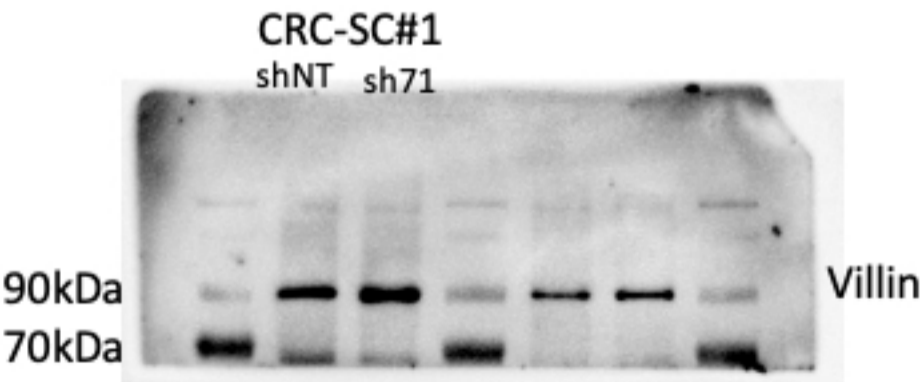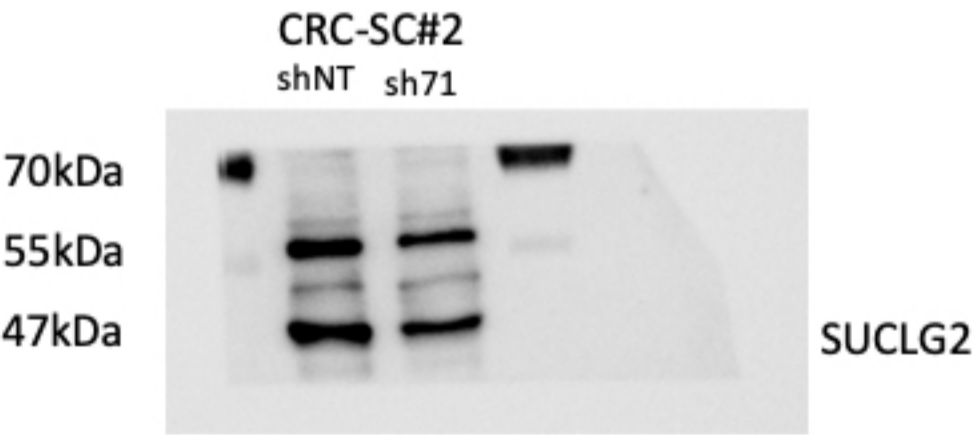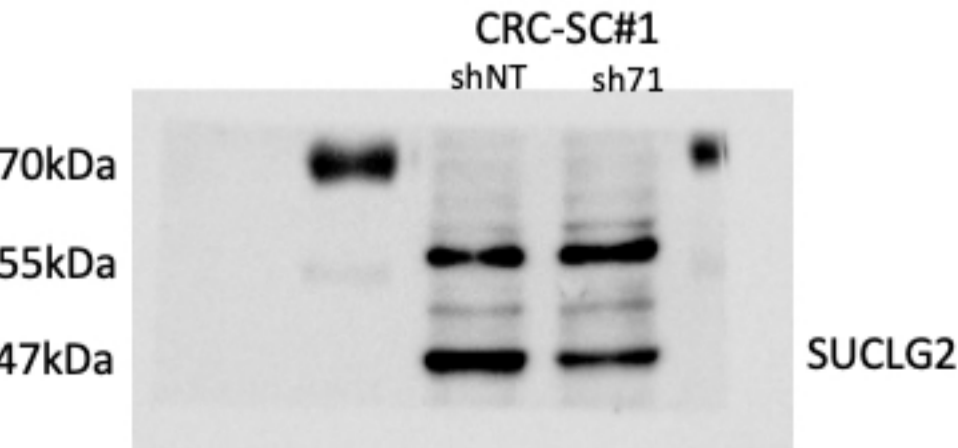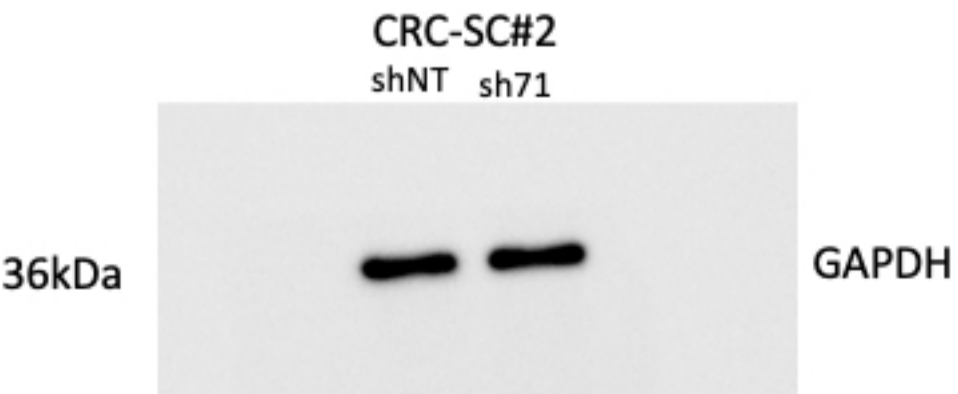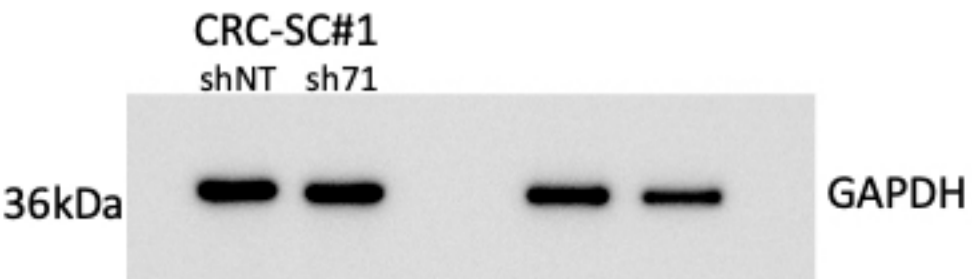

Supplement: Supplementary file 12 — Original Data File [file 41420_2023_1502_MOESM12_ESM.pdf]
