# Supplementary material for: Targeting lysine-specific demethylase 1 (KDM1A/LSD1) impairs colorectal cancer tumorigenesis by affecting cancer cells stemness, motility, and differentiation
Source: Cell Death Discov. 2023 Jun 29;9:201. doi: 10.1038/s41420-023-01502-1 (PMC10310788; doi:10.1038/s41420-023-01502-1)
Supplement: Supplementary file 7 — Supplementary Figure Legends [file 41420_2023_1502_MOESM7_ESM.docx]

**Supplementary Figure 1.** ORY-1001and GSK2879552 display moderate to low toxicity in CRC cells. Cells were treated with increasing doses of the KDM1A inhibitor ORY-1001 and GSK2879552. After 72 hours, cell viability was assessed through MTT assay, and the dose-response graphs were elaborated using GraphPad Prism 8 software. All values are presented as the mean ± standard deviation of at least 3 independent experiments.

**Supplementary Figure 2.** Gene set enrichment analysis (GSEA). GSEA enrichment score curves of LSD1-silenced (sh71) and control (shNT) CRC-SC#1. ES, enrichment score; NES, normalized enrichment score (permutation type: gene-set, metric for gene ranking: diff of classes, p-value < 0.05, gene set database: c5.go.bp.v2023.1.Hs.symbols).

**Supplementary Figure 3.** KDM1A-silenced CRC-SCs are characterized by significant deregulation of the expression of several non-coding RNAs. Graph displaying TPM of lncRNAs and pseudogenes significantly deregulated between CRC-SC#1 silenced cells (sh71) vs CRC-SC#1 control cells (shNT)

**Supplementary Figure 4.** STRING Protein-protein interaction network of deregulated protein in *KDM1A-silenced CRC-SCs.* Significantly deregulated proteins between CRC-SC#1 silenced cells(sh71) vs CRC-SC#1 control cells (shNT) (red color, upregulated proteins; blue color, downregulated proteins). Line thickness indicates the strength of data support (confidence)(**A**).

**Supplementary Figure 5.** KDM1A knockdown in CRC-SC#1 does not compromise mitochondrial respiration and function. Illustrative Oroboros Oxygraphs measuring oxygen concentration (**A**). Representative tracing of high-resolution respirometry to quantify oxygen flux in the routine state (R), in the leakage state (L), after the addition of the ATP synthetase inhibitor oligomycin, and after the addition of the uncoupler of oxidative phosphorylation, FCCP, to quantify maximum respiratory capacity (E) (**B**). The last stage considers the oxygen flux independent from complex I, obtained by the addition of rotenone (ROX Rot). All data are expressed as specific flux, i.e., oxygen consumption normalized to the sample protein content and after non-mitochondrial oxygen flux subtraction (ROX). Histogram showing leakage state of the respiratory chain (**C**), oxygen consumption linked to ATP production (**D**), and reserve respiratory capacity (**E**). All data are expressed as mean ± SEM from at least 3 independent experiments. Mitochondrial membrane potential depolarization evaluation by JC-1 staining (**F**) and histogram showing quantification of red/green, fluorescent ratio as fold change relative to control (**G**). Representative images of Mitotracker-Red of transduced CRC-SC#1 (**H**).

**Supplementary Figure 6.** Integration of transcriptomic and proteomic data revealing that the two techniques are identifying complementary fragments more than different aspects of the biological question. Common DEGs identified with transcriptomics and proteomics, DEGs upregulated in both techniques are displayed in red, downregulate in blue, DEGs not in agreement are indicated in black (**A**). Heatmap displaying unsupervised hierarchical clustering of the genes and proteins which display the same modulation with both techniques (**B**). STRING Protein-protein interaction network of deregulated protein and genes in KDM1A-silenced CRC-SCs. Significant DEGs between CRC-SC#1 silenced cells(sh71) vs CRC-SC#1 control cells (shNT) (red color, upregulated; blue color, down-regulated). Line thickness indicates the strength of data support (confidence)(**C**). Semantic plot of the enriched biological processes (**D**)

**Supplementary Figure 7.** Original western blot membranes
